# Supplementary material for: The novel tRF-23 promotes osteogenic differentiation of hBMSCs and protects against bone loss in ovariectomized mice
Source: Stem Cell Reports. 2025 Oct 2;20(10):102673. doi: 10.1016/j.stemcr.2025.102673 (PMC12790721; doi:10.1016/j.stemcr.2025.102673)
Supplement: Document S1. Figures S1–S4 and Table S1 [file mmc1.pdf]

**Stem Cell Reports, Volume *20***

## Supplemental Information

# The novel tRF-23 promotes osteogenic differentiation of hBMSCs and protects against bone loss in ovariectomized mice

**Haichun Liao, Wen Li, Lin Xu, Chao Zhao, Xingnuan Li, Jianjun Xiong, and Tao Wang**

# Supplemental Information

## The novel tRF-23 promotes osteogenic differentiation of hBMSCs and protects against bone loss in ovariectomized mice

**Haichun Liao, Wen Li, Lin Xu,**

**Chao Zhao, Xingnuan Li, Jianjun Xiong, and Tao Wang**

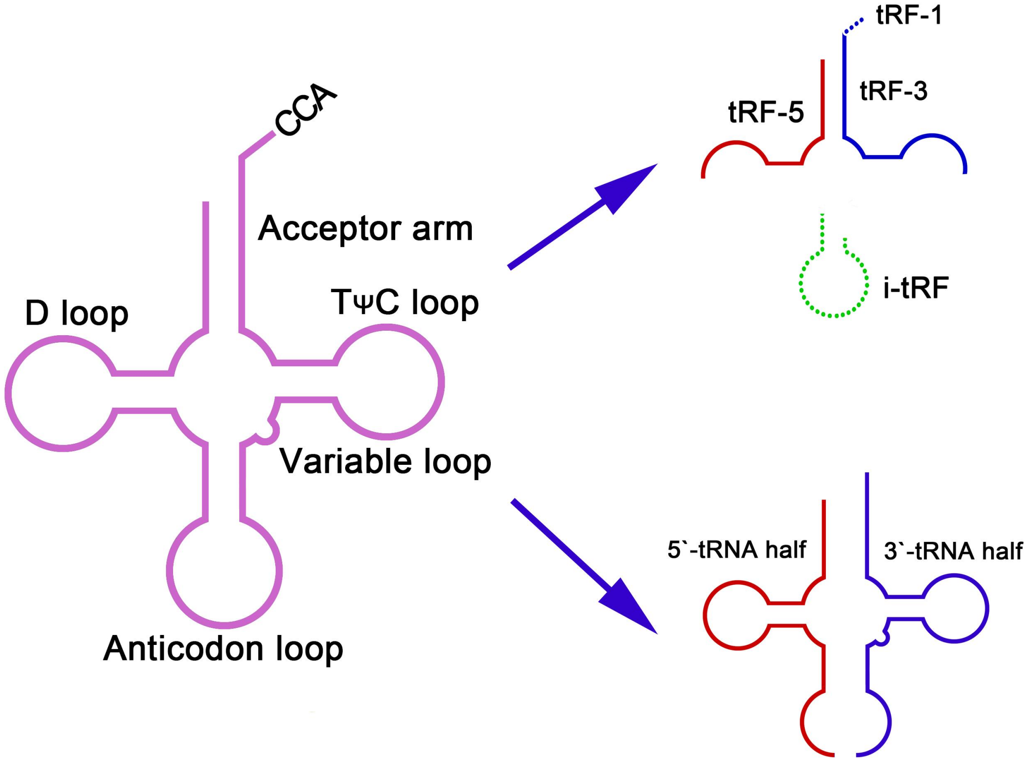


**Figure S1. Different tRFs are derived from pre-tRNAs or mature tRNAs, related to Figure 1**

tRFs are classified as tRF-5 and tRF-3, i-tRFs, tRF-1, and 5′- or 3′-tRNA halves based on their compositions and origins.

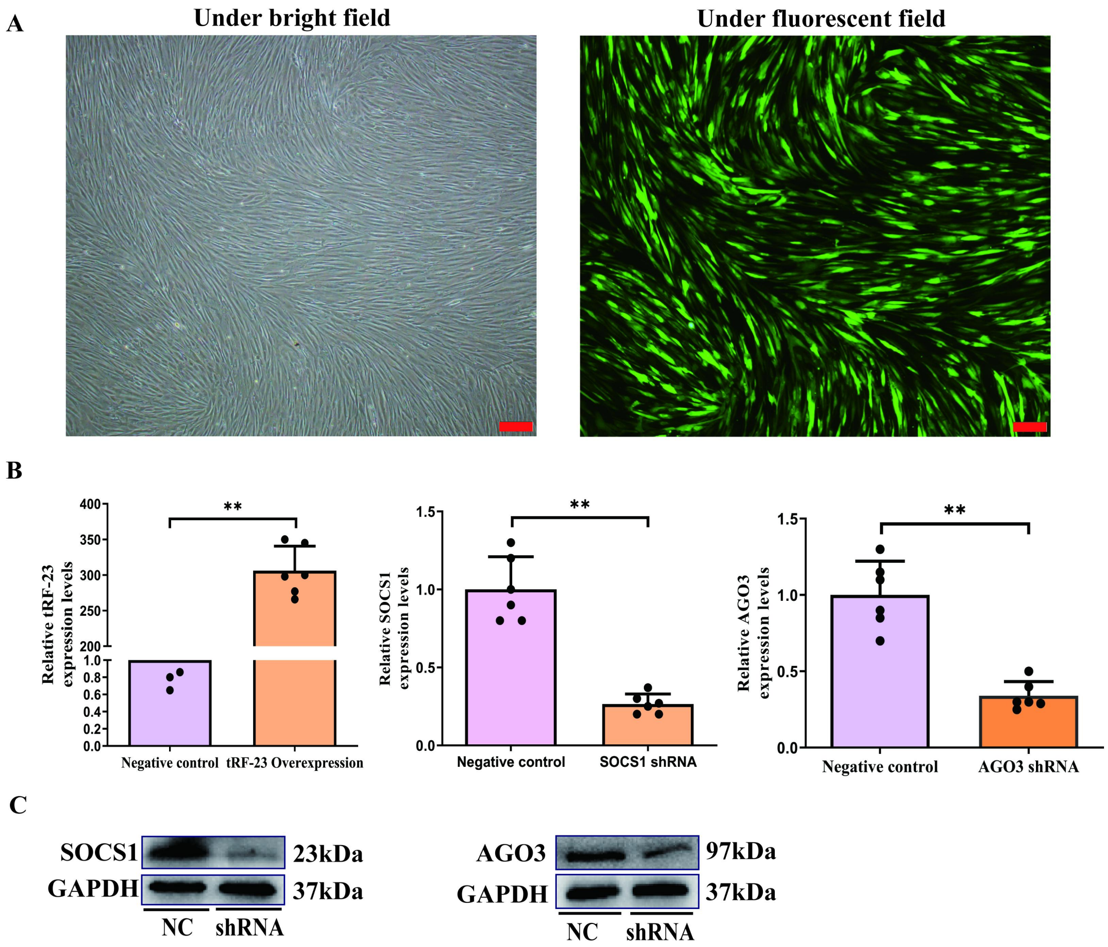


**Figure S2. Efficiency of lentiviral transfection at 6 days post-transfection**

(A) Transfected cells were evaluated using fluorescence and light microscopy, with representative images shown. Scale bar: 100 µm.

(B and C) qPCR and Western immunoblotting were used to evaluate tRF-23, SOCS1, and AGO3 expression after lentiviral transfection. Data are presented as the mean ± SD (n = 3 independent experiments with two technical replicates per independent sample).

Statistical analysis was performed using a two-tailed paired Student's t test. $^{**}P < 0.01$ was considered significant. Note: NC, negative control; shRNA, short hairpin RNA.

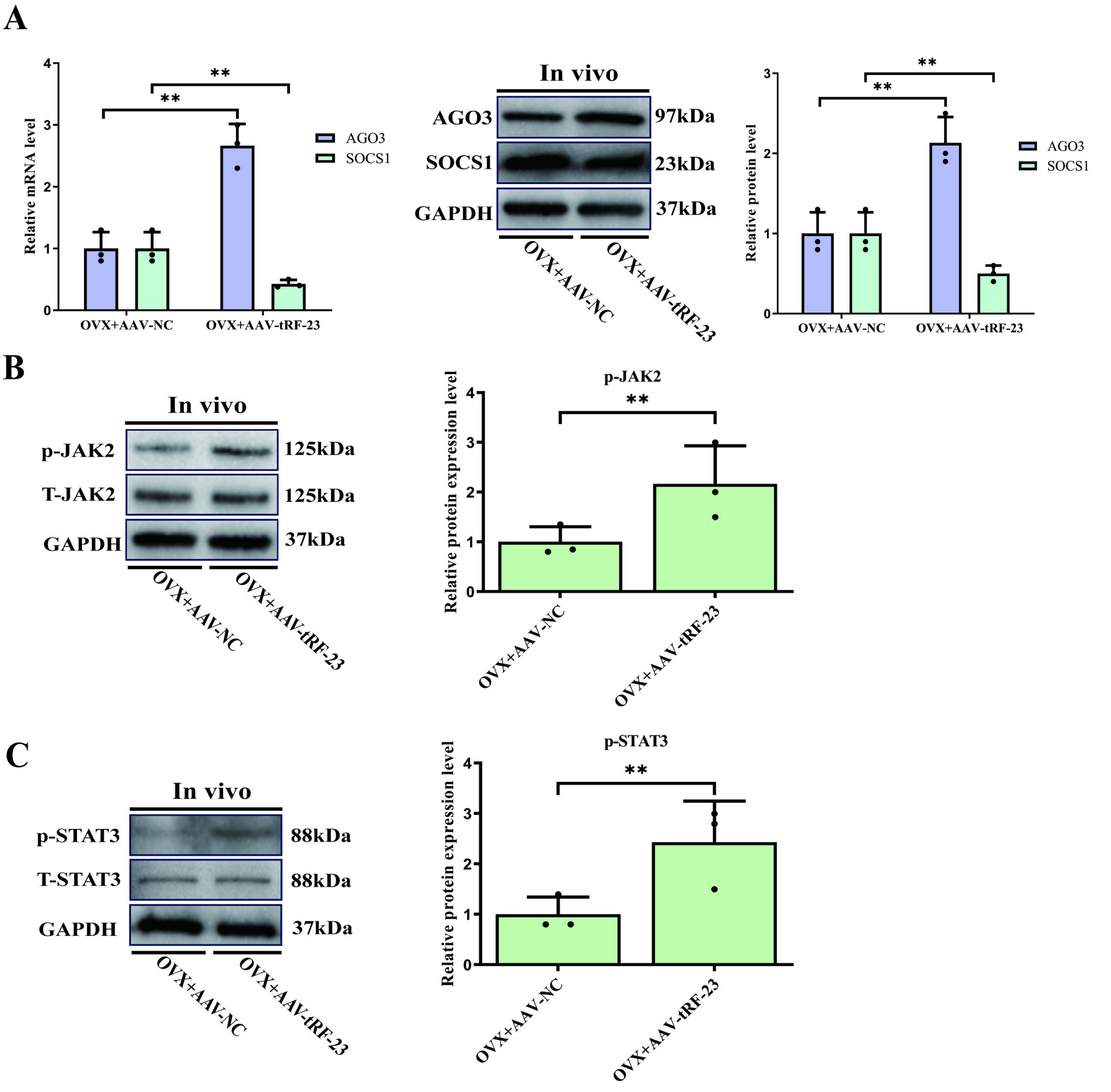


**Figure S3. The effects of intramural injection of AAV9-tRF-23 on the expression of SOCS1, AGO3, and components of the JAK2/STAT3 signaling pathway in mouse femur, related to Figures 6 and 7**

(A)Reduced expression of SOCS1 in the OVX-AAV-tRF-23 mouse model, with increased expression of AGO3 compared with the OVX-AAV-NC group. Data are presented as the mean ± SD (n = 3 independent experiments).

(B and C) AVV-tRF-23 administration to OVX model mice induced increased levels of p-JAK2/STAT3 proteins compared with the OVX-AAV-NC group. Data are presented as the mean ± SD (n = 3 independent experiments).

Statistical analysis was performed using a two-tailed paired Student's t test. $^{**}P < 0.01$ was considered significant. Note: NC, negative control.

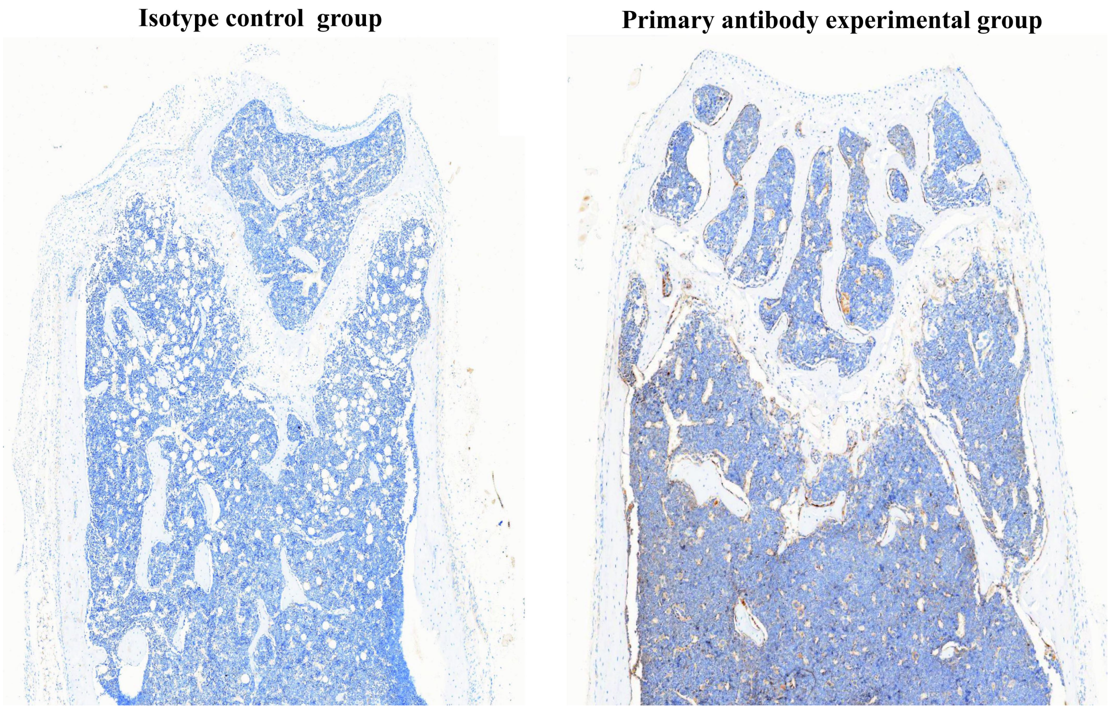


**Figure S4. Non-immune immunoglobulins of the same isotype as the primary antibodies used as negative controls, related to Figure 7**

The results confirmed that there was no nonspecific staining in the isotype control groups, verifying the specificity of the primary antibody signals.

## Table S1. Primer sets used in this study.

| Gene symbol | Forward primers | Reverse primers |
|---|---|---|
| RUNX2 | 5′-GGACGAGGCAAGAGTTTCACC-3′ | 5′-GGTTCCCGAGGTCCATCTACT-3′ |
| OCN | 5′-TGAGAGCCCTCACACTCCTC-3′ | 5′-CGCCTGGGTCTCTTCACTAC-3′ |
| ALP | 5′-CCCCGTGGCAACTCTATCTTT-3′ | 5′-GCCTGGTAGTTGTTGTGAGCATAG-3′ |
| SOCS1 | 5′-GAGCTGCTGGAGCACTACG-3′ | 5′-AGGGGAAGGAGCTCAGGTAG-3′ |
| AGO3 | 5′-ACATGCGAGGGAAACAATTC-3′ | 5′-ACATGGGCTCTACGCTGTCT-3′ |
| β-actin | 5′-GCGAGAAGATGACCCAGATCATGT-3′ | 5′-TACCCCTCGTAGATGGGCACA-3′ |
| tRF-23 | 5′-CTCAACTGGTGTCGTGGAGTCGGCAATTCAGTTGAGGGCGGTGA-3′ | 5′-ACACTCCAGCTGGGTAGGATTCGGCGCTCTC-3′ |
| U6 | 5′-CTCGCTTCGGCAGCACA-3′ | 5′-AACGCTTCACGAATTTGCGT-3′ |
