# Supplementary material for: The novel tRF-23 promotes osteogenic differentiation of hBMSCs and protects against bone loss in ovariectomized mice
Source: Stem Cell Reports. 2025 Oct 2;20(10):102673. doi: 10.1016/j.stemcr.2025.102673 (PMC12790721; doi:10.1016/j.stemcr.2025.102673)
Supplement: Document S2. Article plus supplemental information [file mmc2.pdf]

Stem Cell Reports

Article

OPEN ACCESS

# The novel tRF-23 promotes osteogenic differentiation of hBMSCs and protects against bone loss in ovariectomized mice

Haichun Liao,[1,2] Wen Li,[3] Lin Xu,[2] Chao Zhao,[4] Xingnuan Li,[2,*] Jianjun Xiong,[2,*] and Tao Wang[2,5,*]

[1]School of Health Science and Engineering, University of Shanghai for Science and Technology, Shanghai 200093, China

[2]Jiangxi Provincial Key Laboratory of Cell Precision Therapy, School of Basic Medical Sciences, Jiujiang University, Jiujiang 332005, China

[3]Department of Hematology and Oncology, Affiliated Hospital of Jiujiang University, Jiujiang 332005, China

[4]Department of Thyroid and Breast Surgery, Affiliated Hospital of Jiujiang University, Jiujiang 332005, China

[5]Lead contact

*Correspondence: 6140074@jju.edu.cn (X.L.), xiongjj1975@163.com (J.X.), comwangtaocom@163.com (T.W.)

https://doi.org/10.1016/j.stemcr.2025.102673

## SUMMARY

The pathogenesis of osteoporosis is closely related to the impaired human bone marrow-derived stromal cells (hBMSCs) osteogenic differentiation. No studies to date, however, have established whether tRNA-derived fragments (tRFs) can influence osteogenic differentiation of hBMSCs or the onset of osteoporosis. Here, tRF-23 was found to control hBMSC osteogenesis through its ability to target suppressor of cytokine signaling 1 (SOCS1) via the Janus kinase 2/signal transducer and activator of transcription 3 (JAK2/STAT3) signaling pathway. tRF-23 was then further established as a potential target for efforts to protect against bone loss and marrow adipose tissue (MAT) accumulation in osteoporotic model mice, and its molecular mechanism was also verified *in vivo*. Together, these results suggest a model in which tRF-23 can protect against bone loss induced by ovariectomized (OVX) through the augmentation of hBMSC osteogenesis, providing a foundation for characterizing the pathogenesis of osteoporosis and seeking new therapeutic targets for this disruptive condition.

## INTRODUCTION

Osteoporosis is a systemic bone disease resulting in the weakening of bone structures, a reduction in overall bone mass, bone microstructural destruction, an increase in the brittleness of bones, and a greater overall risk of fracture (Black and Rosen, 2016). As the global population continues to age, osteoporosis is increasingly emerging as a prevalent and costly disease globally (Johnston and Dagar, 2020). Osteoporosis cases are broadly classified into primary and secondary osteoporosis, with primary cases being further subclassified into postmenopausal and senile osteoporosis cases, the former of which are more common (Gao and Zhao, 2023). Patients suffering from osteoporosis are prone to bone fractures, and death can occur in severe cases. Osteoporosis also exacts an immense toll on the overall health and quality of life of affected patients, imposing a major burden on these individuals and society as a whole (Marcellusi et al., 2020).

Human bone marrow-derived stromal cells (hBMSCs) exhibit multipotent differentiation capabilities such that they can develop into osteoblasts, adipocytes, and chondroblasts, with hBMSC-derived osteoblasts being particularly important in the context of bone formation, maintenance, and reconstruction (Su et al., 2018). In prior reports, hBMSC osteogenesis has been closely linked to the etiological development of primary osteoporosis (Xia et al., 2023). As such, efforts to promote stem cell osteogenesis represent an attractive approach to treating osteoporosis.

Advanced sequencing technologies have fueled the recognition that tRNA loci in the genome can also produce small non-coding RNAs (sncRNAs), among which transfer RNA (tRNA)-derived fragments (tRFs), also known as tRNA-derived small RNAs (tsRNAs), have emerged as a particular focus of research interest (Keam and Hutvagner, 2015). Based on the specific tRNA loci from which they originate, these tRFs have been assigned to six different classes (Figure S1) (Wang et al., 2020). While angiogenin-mediated anticodon site tRNA cleavage gives rise to 5′- and 3′-tRNA halves, tRF-5 fragments are generated by the cleavage of mature tRNAs at the D-loop or anticodon step, and tRF-3 fragments arise from similar T-loop or anticodon stem cleavage. Moreover, tRF-1 fragments are produced from the 3′ end fragments of primary tRNAs, and internal tRFs (i-tRFs), arise from internal fragments of mature tRNAs.

The expression of tRFs has been studied in many different biological settings, prompting growing interest in the exploration of how these transcripts influence both normal physiological processes and disease incidence. Particular tRFs have been found to inhibit oncogenesis (Zhou et al., 2024), control ribosomal biogenesis in cancer cells (Kim et al., 2020), shape paternal epigenetic inheritance (Zeng et al., 2022), and enable long terminal repeat (LTR)-retrotransposon control through assorted mechanisms (Schorn et al., 2017). Other studies have also highlighted the promise of tRFs as biomarkers that can guide the diagnosis of conditions including systemic lupus erythematosus nephritis, Alzheimer's disease, cancer, and


Published by Elsevier Inc. on behalf of International Society for Stem Cell Research.
This is an open access article under the CC BY license (http://creativecommons.org/licenses/by/4.0/).

Parkinson's disease (Jia et al., 2020; Weng et al., 2022). However, whether tRFs influence the osteogenic differentiation of hBMSCs or the occurrence of osteoporosis is not known.

Here, the novel tRF-23-V2Y8L981DV, encoded on chromosome 1 (abbreviated as tRF-23, i-tRF type), was identified as a novel regulator of hBMSC osteogenesis through its ability to target suppressor of cytokine signaling 1 (SOCS1) through the Janus kinase 2/signal transducer and activator of transcription 3 (JAK2/STAT3) signaling axis. Overexpressing tRF-23 via the injection of recombinant adeno-associated virus serotype 9-tRF-23 (AAV9-tRF-23) in the bone marrow was found to enhance bone formation and reduce bone marrow fat levels in ovariectomized (OVX) mice. These findings offer novel insight into the potential applicability of tRF-23 to osteoporosis management.

## RESULTS

### Evaluation of tRF expression profiles during hBMSC osteogenesis

Initial analyses were conducted to examine the differential expression of tRFs within hBMSCs over the course of osteogenesis by harvesting RNA from these cells on days 0, 7, 14, and 21 of osteogenic differentiation for small RNA sequencing (RNA-seq) performed with the Illumina HiSeq X-ten instrument. Reads 12–50 nucleotides (nt) in length were then filtered with the piRNA (Piwi-interacting RNA) and GtRNA (Genomic tRNA) databases, followed by their alignment with sequences from tRFdb and tRF MINTbase, thereby establishing tRF expression profiles for these samples. The identified tRFs at all time points primarily ranged between 16 and 35 nt long (Figure 1A).

The identification of tRFs differentially expressed (log2FC > 1 and false discovery rate [FDR] < 0.05) over the course of hBMSC osteogenesis was next performed with the EBSeq algorithm, comparing tRFs screened on days 0 vs. 7, 7 vs. 14, and 14 vs. 21. Peak expression levels were observed for most of these tRFs on day 7 (Figure 1B). As such, tRF expression on day 7 of osteogenesis was selected as the main focus. It is well known that differential expression of tRFs is not necessarily associated with biological function. Therefore, we selected four tRFs with significantly increased expression on day 7 of osteogenesis and then knocked down their expression. This showed that knockdown of tRF-23 inhibited the osteogenic differentiation of hBMSCs. Based on these preliminary experiments, tRF-23 was chosen as a target for further analysis (Figure 1C). The expression of tRF-23 during osteogenic differentiation was also analyzed using quantitative reverse-transcription polymerase chain reaction (RT-qPCR), confirming the reliability of the small RNA-seq results (Figure 1D).

### Lentiviral transfection and its effects

To alter the expression of tRF-23, cells stably expressing tRF-23 overexpression, tRF-23 short hairpin RNA (shRNA), or control vectors were established. Similarly, shRNA vectors were prepared for SOCS1 and argonaute 3 (AGO3). After puromycin selection, these cells exhibited good growth and strong green fluorescent protein (GFP) fluorescence consistent with their having been stably transfected (Figure S2A). Successful transfection was confirmed by RT-qPCR, which revealed that tRF-23 was overexpressed by 300-fold as compared to negative control (NC) cells (Figure S2B). The tRF and miRNA lentiviruses were constructed based on the same principle; the efficiency of tRF-23 knockdown cannot be detected via qPCR. SOCS1 and AGO3 knockdown efficiency was further demonstrated via qPCR and western immunoblotting (Figures S2B and S2C), both of which were downregulated 2- to 3-fold relative to appropriate controls.

### tRF-23 regulates hBMSC osteogenic differentiation

To examine the impact of tRF-23 on the process of osteogenesis, the differentiation of hBMSCs in which this tRF had been overexpressed or knocked down was next assessed on day 14 of culture in osteogenic medium. Alizarin red S (ARS) staining on day 14 revealed that overexpressing tRF-23 led to an increase in staining intensity as compared to control cells, consistent with the enhancement of matrix mineralization. Conversely, tRF-23 silencing led to a reduction in mineralization-related staining intensity (Figure 2A).

In line with the aforementioned results, alkaline phosphatase (ALP) staining performed on day 14 of osteogenesis revealed that overexpressing tRF-23 led to stronger ALP staining (blue-violet cells), whereas the knockdown of tRF-23 led to weaker positive staining (Figure 2B). These results were also confirmed through the quantification of matrix mineralization and ALP activity (Figure 2B).

To expand on these results, qPCR and western blotting were next used to probe the expression of osteogenic markers, namely, Runt-related transcription factor 2 (RUNX2), ALP, and osteocalcin (OCN). Overexpressing led to significantly enhanced OCN and RUNX2 expression, whereas the silencing of this tRF yielded opposing effects (Figures 2C–2F).

### tRF-23 directly targets SOCS1

Using the miRanda and RNAhybrid databases, a putative site for tRF-23 binding was detected within the 3′-untranslated region (3′-UTR) of SOCS1 (Figure 3A). Validating this binding site, luciferase reporter activity was found to be reduced by 40% in response to tRF-23 transfection when expressing reporter vectors with a wild-type (WT) version of this SOCS1 3′-UTR binding site, while the same was

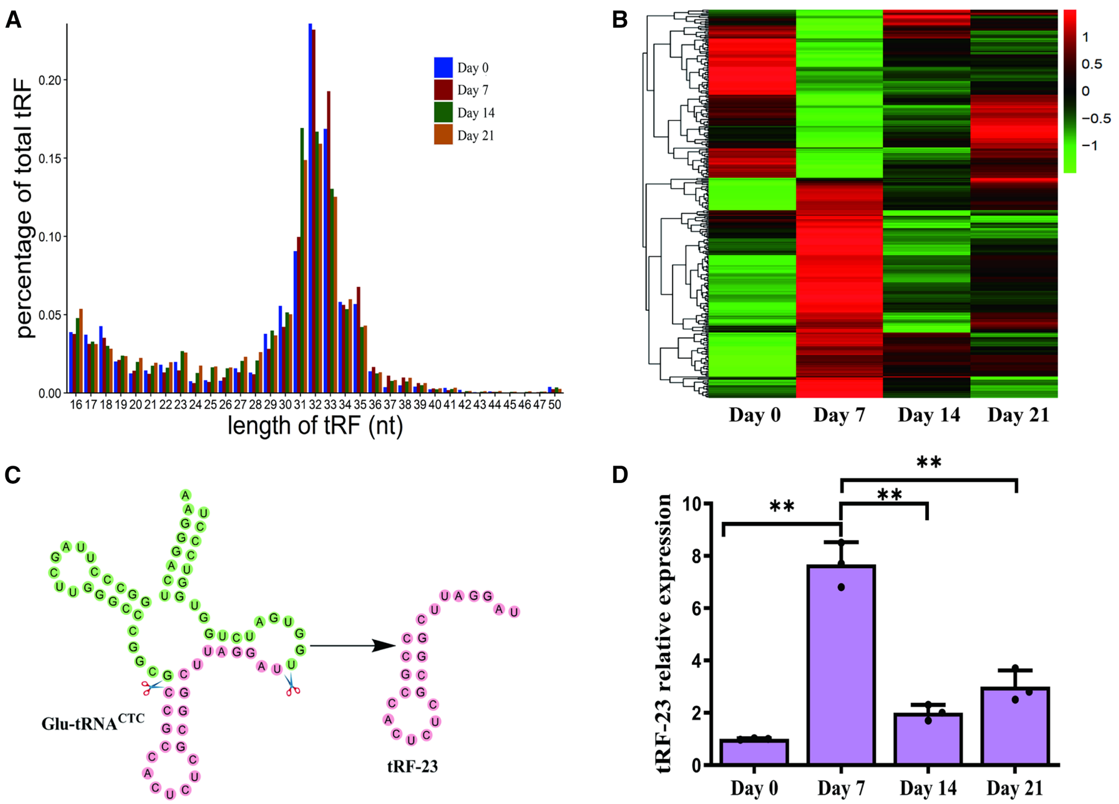


**Figure 1. Analyses of tRFs expression during hBMSCs osteogenesis**

(A) tRF read distributions during osteogenesis, with tRF length variance reported as percentages of the total tRF reads.

(B) Differentially expressed tRFs are presented in a heatmap.

(C) Differentially expressed tRFs were analyzed in detail on day 7, with a focus on tRF-23 resulting from the digestion of tRNA-Glu-CTC.

(D) Differential tRF-23 expression was analyzed over the course of hBMSC osteogenesis. Data are presented as the mean ± SD ($n$ = 3 independent experiments). Statistical analysis was performed using a two-tailed paired Student's t test. $^{**}p < 0.01$ was considered significant.

not observed if this binding site had been mutated (Figure 3B). The effects of overexpression and knockdown of tRF-23 on SOCS1 expression on day 14 of osteogenic differentiation were then analyzed (Figures 3C and 3D). The effects of SOCS1 on hBMSCs osteogenesis were next evaluated by knocking down this gene in these cells. This SOCS1 silencing markedly increased osteogenic differentiation on day 14 (Figure 3E), and this was further supported by significantly reduced RUNX2, ALP, and OCN expression (Figures 3F and 3G).

### AGO3 serves as a mediator of the targeting of SOCS1 by tRF-23

Comprehensive identification of RNA-binding proteins by mass spectrometry (ChIRP-MS) analyses were next conducted in which AGO3 was screened out as a protein binding partner for tRF-23, and the mass spectrum for AGO3 was also identified (Figures 4A and 4B). To confirm the ability of AGO3 and tRF-23 to interact, an RNA immunoprecipitation (RIP) approach was next implemented, ultimately validating this interaction and then a qPCR analysis of the products of immunoprecipitation also confirmed higher levels of tRF-23 expression in the AGO3 group relative to samples precipitated with control IgG (Figures 4C and 4D). When AGO3 was knocked down in cells overexpressing tRF-23 or in which SOCS1 was knocked down, an increase in SOCS1 expression was evident relative to control cells (Figures 4E and 4F), consistent with the ability of AGO3 to mediate tRF-23 targeting of SOCS1.

### tRF-23 targets SOCS1 to control hBMSCs osteogenesis through the regulation of JAK2/STAT3 activity

To determine whether tRF-23 influences JAK2/STAT3 signaling activity during osteogenesis, western immunoblotting was next used to measure phosphorylated JAK2 (p-JAK2) and phosphorylated STAT3 (p-STAT3) levels in stably transduced hBMSCs during osteogenesis. While overexpressing tRF-23 led to increases in both p-JAK2 and

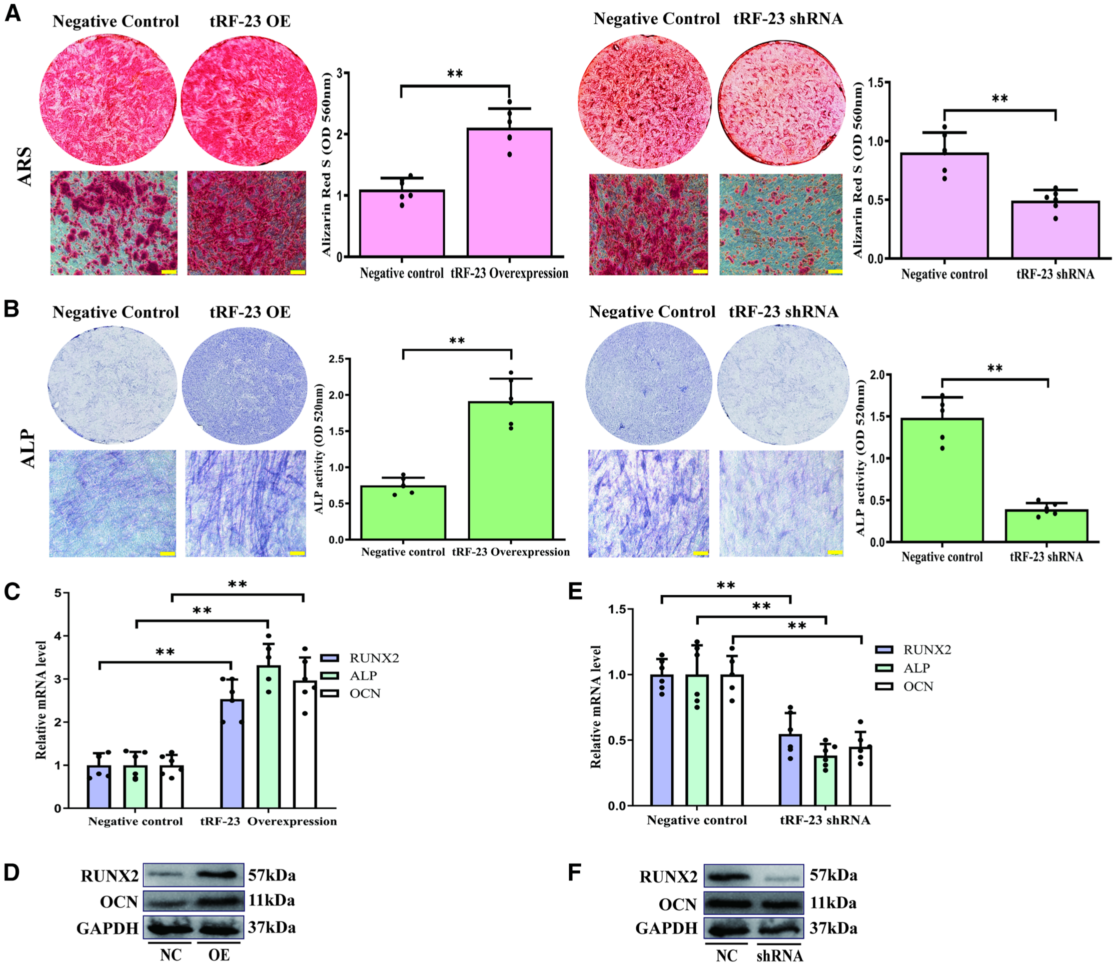


**Figure 2. tRF-23 modulates hBMSC osteogenic differentiation at 14 days**

(A and B) Overexpressing and silencing tRF-23 respectively enhanced/reduced matrix mineralization and the frequency of positive blue-violet positive cell staining. Scale bars: 50 μm. Matrix mineralization and ALP activity were quantified based on absorbance. Barplot shows the mean ± SD from 3 independent experiments with two technical replicates per independent sample.

(C and D) RUNX2, OCN, and ALP levels in the tRF-23 overexpression group were analyzed via qPCR and western blotting. Barplot shows the mean ± SD from 3 independent experiments with two technical replicates per independent sample.

(E and F) RUNX2, OCN, and ALP levels in the tRF-23 knockdown group via qPCR and western blotting. Barplot shows the mean ± SD from 3 independent experiments with two technical replicates per independent sample.

Statistical significance was determined using a two-tailed paired Student's t test. $^{**}p < 0.01$ was considered significant. NC, negative control; OE, overexpression; shRNA, short hairpin RNA.

p-STAT3 levels, the opposite was true when this tRF was knocked down (Figures 5A and 5B). To suppress JAK2/STAT3 phosphorylation in these tRF-23-overexpressing cells, they were treated with the small molecule JAK2/STAT3 inhibitor AG490 (100 nM; Selleck, cat no. S7259). This treatment weakened the osteogenic differentiation of hBMSCs overexpressing tRF-23 relative to control tRF-23-overexpressing hBMSCs (Figures 5C–5E). These findings confirmed that the SOCS1/JAK2/STAT3 signaling axis functions downstream of tRF-23.

### Injection of AAV9-tRF-23 into the bone marrow cavity protects against bone loss and marrow adipose tissue accumulation in a mouse model of OVX-induced osteoporosis

The ability of tRF-23 overexpression to protect against osteoporosis was next evaluated in detail. To that end, a murine model of osteoporosis was established by ovariectomizing two-month-old female C57BL/6J mice, followed by the injection of AAV9-tRF-23 within the bone marrow cavity in a subset of these mice (Figure 6A). The femurs of the mice

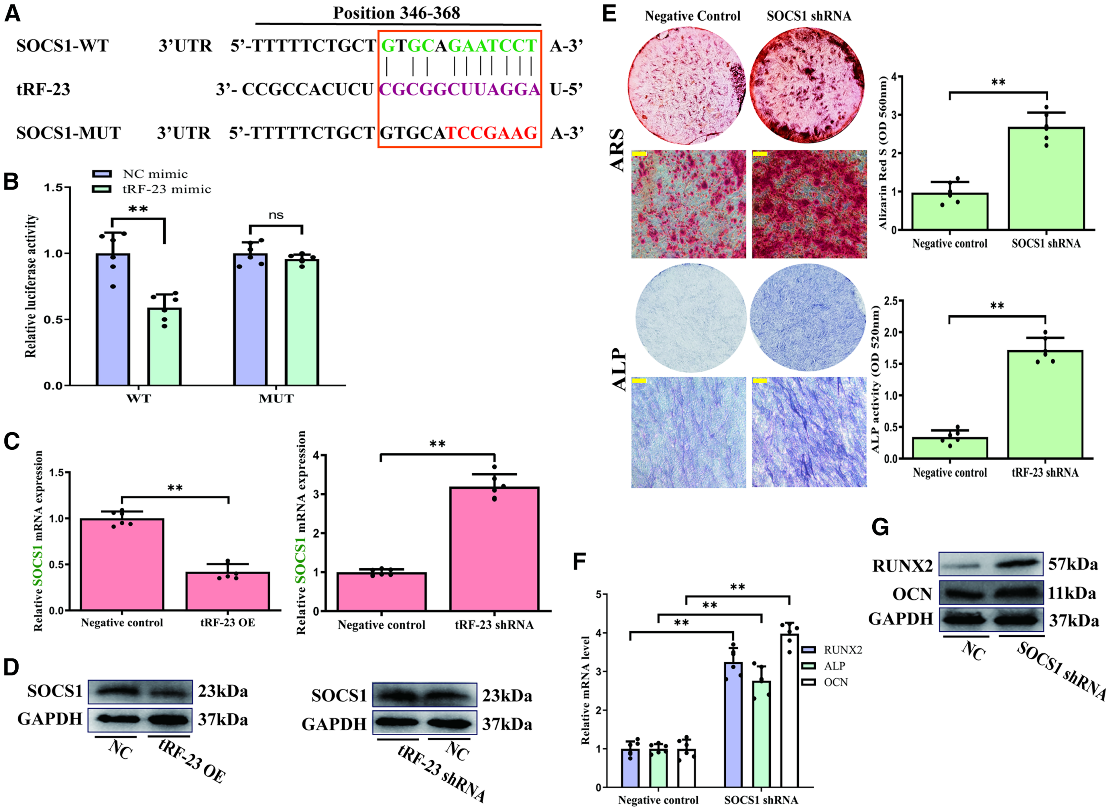


**Figure 3. The SOCS1 3′-UTR is a tRF-23 target**

(A) A schematic of the predicted tRF-23 binding site in the SOCS1 3′-UTR, with residues that were selectively mutated highlighted in red.
(B) Luciferase activity analyses indicated that transfection with tRF-23 reduced WT but not mutated SOCS1 3′-UTR reporter construct luciferase activity by 40%. Data are presented as mean ± SD ($n$ = 3 independent experiments, each with two technical replicates per independent sample).
(C and D) The effects of tRF-23 on SOCS1 expression on day 14 of hBMSC osteogenesis were analyzed. Data are presented as the mean ± SD ($n$ = 3 independent experiments with two technical replicates per independent sample).
(E) SOCS1 knockdown enhanced matrix mineralization and positive (blue-violet) staining. Scale bars: 50 μm. Matrix mineralization and ALP activity were quantified based on absorbance. Data are presented as the mean ± SD ($n$ = 3 independent experiments with two technical replicates per independent sample).
(F and G) RUNX2, OCN, and ALP levels on day 14 of hBMSC osteogenesis were detected via qPCR and western blotting. Data are presented as the mean ± SD ($n$ = 3 independent experiments with two technical replicates per independent sample).
Statistical analysis was performed using paired two-tailed Student's t tests. $^{**}p < 0.01$ was considered significant. NC, negative control; OE, overexpression; shRNA, short hairpin RNA; ns, not significant.

showed marked green fluorescence, indicating the infectivity of AAV, and tRF-23 expression levels in the bone were also confirmed by RT-qPCR (Figure 6B). Micro-CT images of the femurs of these mice revealed that OVX-AAV9-tRF-23 treatment was associated with larger amounts of trabecular bone as compared to that observed in OVX-AAV9-NC mice (Figure 6C). The distal femur was then selected as the region of interest (ROI), as shown in Figure 6C. Further, quantitative micro-CT analyses also revealed clear changes in trabecular bone volume (Tb. BV/TV), trabecular thickness (Tb. Th), trabecular number (Tb. N), and trabecular separation (Tb. Sp) following AAV9-tRF-23 treatment (Figures 6D–6G). Alizarin-3-methyliminodiacetic acid dual staining also revealed a significant increase in the mineral apposition rate (MAR) in OVX model mice following AAV9-tRF-23 treatment (Figures 6H and 6I). AAV9-tRF-23 also reduced marrow adipose tissue (MAT) deposition and enhanced bone formation relative to the OVX-AAV9-NC mice. Moreover, AAV9-tRF-23 led to reduced MAT deposition (Figures 7A and 7B) and increased bone formation (Figures 7C and 7D), as indicated by HE and OCN staining, respectively. Although tRF-23 treatment did not fully restore trabeculae bone density to those of the Sham group, it significantly alleviated osteoporosis.

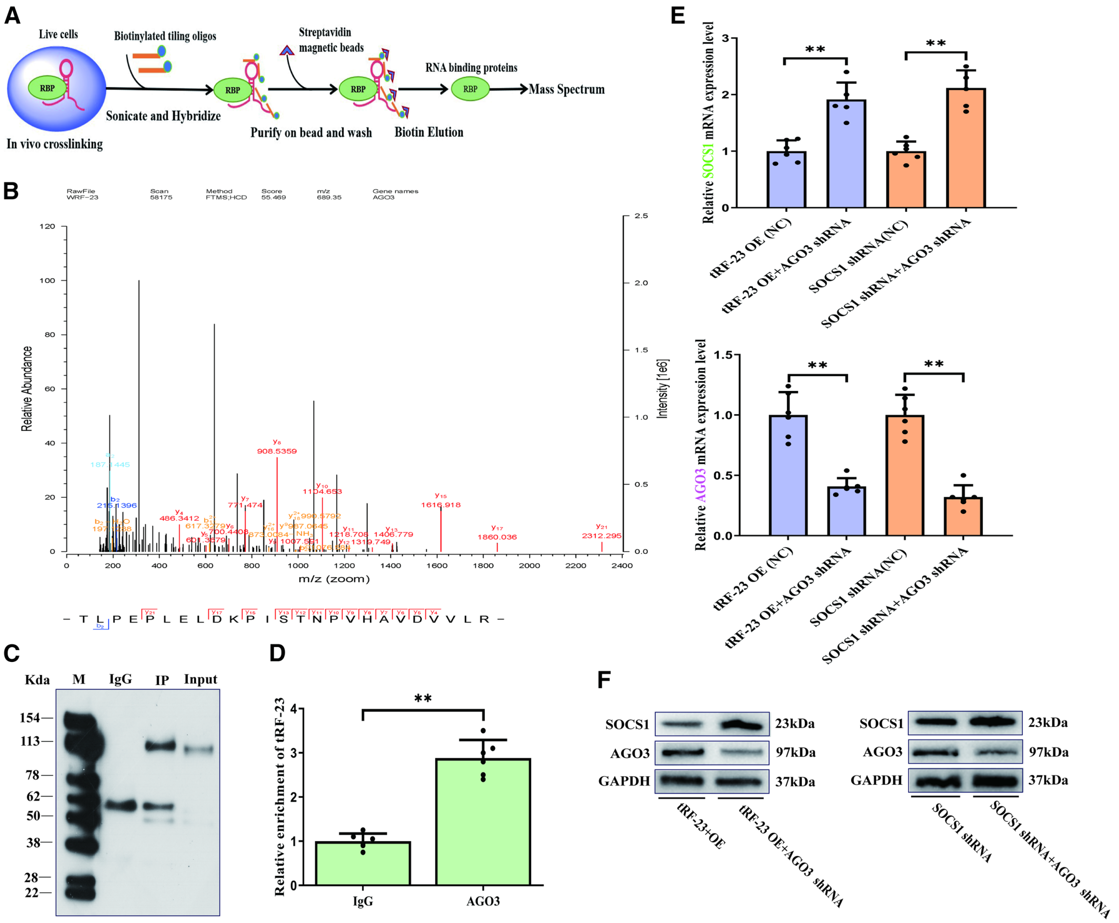


**Figure 4. AGO3 mediates the targeting of SOCS1 by tRF-23**

(A) ChIRP-MS assay schematic overview.

(B) High-resolution MS/MS spectra for tRF-23 oligos and AGO3 cross-linked peptide pairs.

(C) tRF-23 and AGO3 interactions in hBMSCs were analyzed in RIP assays. IgG: negative control; input: positive control; IP: immunoprecipitation.

(D) tRF-23 expression was quantified in IP products by qPCR. Data are presented as the mean ± SD ($n = 3$ independent experiments with two technical replicates per independent sample).

(E and F) SOCS1 and AGO3 levels on day 14 of hBMSCs osteogenesis were detected via qPCR and western blotting. Data are presented as the mean ± SD ($n = 3$ independent experiments with two technical replicates per independent sample).

Statistical analysis was performed using a two-tailed paired Student's t test. $^{**}p < 0.01$ was considered significant. NC, negative control; OE, overexpression; shRNA, short hairpin RNA.

## The effects of intramural injection of AAV9-tRF-23 on the expressions of SOCS1 and AGO3 and the JAK2/STAT3 signaling pathway in mouse femurs

The associations among SOCS1, AGO3, and the JAK2/STAT3 signaling pathway in osteoporosis progression were then evaluated. AAV9-tRF-23 was injected into OVX mice. The mice were euthanized 12 weeks after the injection, and their femurs were collected for analysis. As depicted in Figure S3A, compared with the OVX-AAV-NC group, the expression level of SOCS1 in the OVX-AAV-tRF-23 mouse model was reduced, while that of AGO3 was increased. Western blotting was then used to assess the levels of proteins related to the JAK2/STAT3 signaling pathway. As illustrated in Figures S3B and S3C, following AVV-tRF-23 injection in OVX mice, the protein levels of p-JAK2 and p-STAT3 were increased in the OVX-AAV-tRF-23 group compared with those in the OVX-AAV-NC group. These results are consistent with those of the *in vitro* cell experiments.

## DISCUSSION

The progressive aging of the global population has led to the emergence of osteoporosis as an important clinical

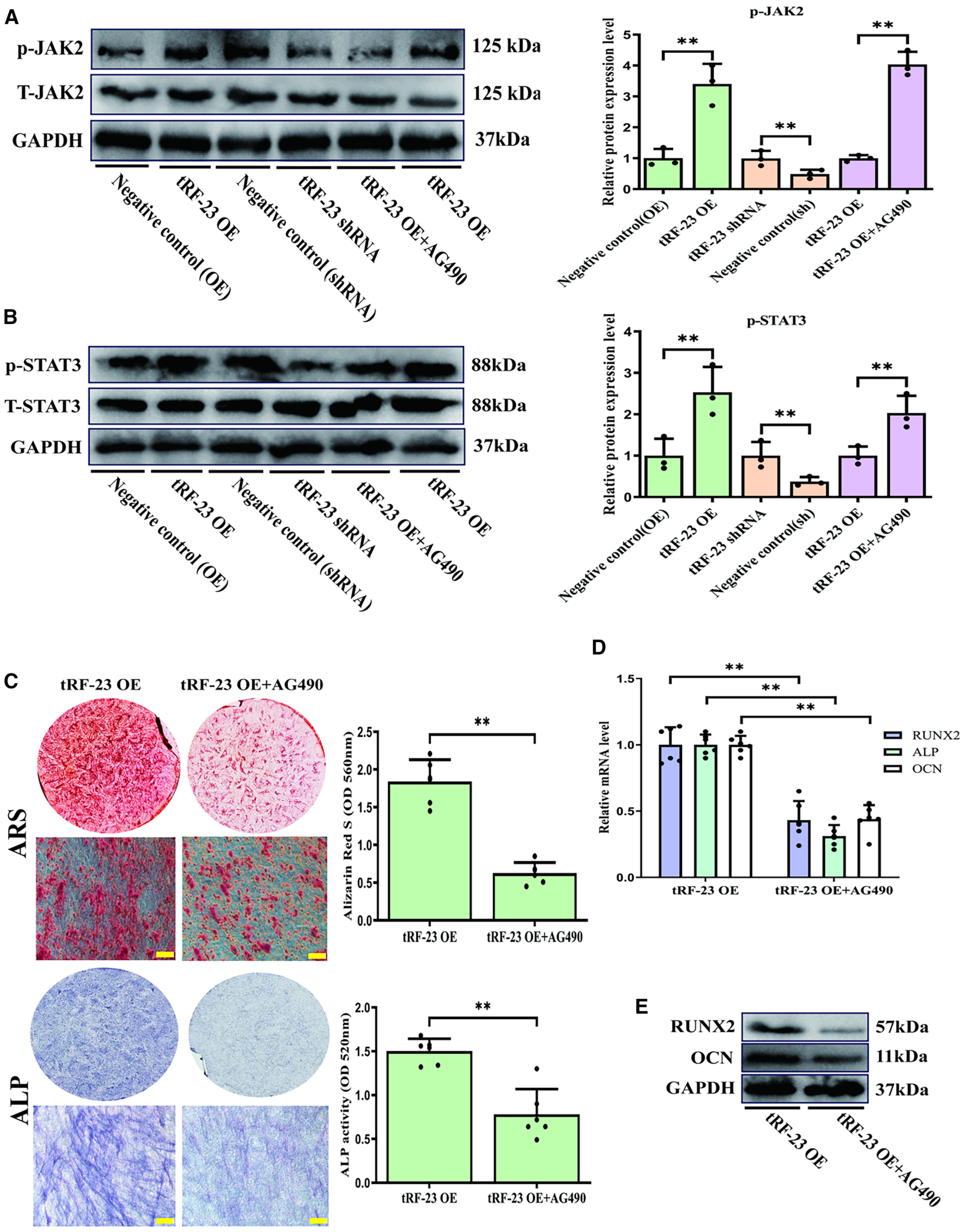


**Figure 5. tRF-23 regulates the osteogenesis of hBMSCs by targeting SOCS1 and regulating JAK2/STAT3 activity**

(A and B) Overexpressing and knocking down tRF-23 respectively led to higher and lower levels of p-JAK2/STAT3. JAK2/STAT3 inhibitor treatment also significantly reduced levels of p-JAK2/STAT3 relative to tRF-23 overexpression in the absence of such inhibition (p-JAK2, normalized to total JAK2; p-STAT3, normalized to total STAT3). Data are presented as the mean ± SD ($n = 3$ independent experiments with two technical replicates per independent sample).

*(legend continued on next page)*

challenge. The two main conventional classes of osteoporosis therapies include anabolic drugs that promote enhanced bone formation and antiresorptive drugs that serve to limit bone resorption (Rachner et al., 2011). These drugs, however, are characterized by the potential for serious adverse reactions and poor long-term curative efficacy. Several reports have demonstrated that osteoporosis is closely linked to the impaired capacity of hBMSCs for osteogenic differentiation (Xia et al., 2023). Alternative therapeutic strategies focused on enhancing hBMSCs osteogenesis may thus provide unprecedented and more efficacious opportunities for the management of osteoporosis.

tRFs are novel sncRNAs that play a diverse range of biological roles in assorted contexts (Zhang et al., 2023). For example, tsRNA-10277-loaded BMSC exosomes were found to promote osteogenic differentiation in dexamethasone-treated BMSCs (Fang et al., 2020) and M1 macrophage-derived extracellular vesicles containing tsRNA-5006c increased osteogenic differentiation in aortic valve interstitial cells through regulation of mitophagy (Xia et al., 2022). Little is known, however, regarding the roles that tRFs play during hBMSC osteogenesis. Here, tRFs expression levels were preliminary profiled in the context of hBMSC osteogenic differentiation, revealing that these tRFs were primarily 16–35 nt long, consistent with their not being the result of random degradation (Figure 1A). Screened differentially expressed tRFs were further assessed at different time points and found to peak on day 7 (Figure 1B), which is a critical stage that determines the fate of osteogenic differentiation. These tRFs were thus considered to potentially serve as key osteogenic regulators and tRF-23 was selected as a target for follow-up research (Figures 1C and 1D). The question of whether tRF-23 ultimately influences osteogenic differentiation of hBMSCs may be asked. It was found that overexpression of tRF-23 enhanced osteogenic differentiation of hBMSCs at 14 days, while its knockdown yielded the opposite outcome (Figure 2), suggesting that tRF-23 is a key regulator of hBMSCs osteogenesis.

The specific mechanisms through which tRF-23 can control hBMSCs osteogenesis have yet to be firmly established, with several studies having demonstrated that some tRFs may function in a manner analogous to miRNAs (Venkatesh et al., 2016; Martinez et al., 2017). The more thorough characterization of how tRF-23 regulates osteogenesis may help better inform the treatment of osteoporosis. Possible tRF-23 target genes were therefore analyzed, leading to the validation of SOCS1 as one such target gene. The direct interaction between tRF-23 and the SOCS1 3′-UTR was then confirmed in a luciferase reporter assay (Figures 3A and 3B). The levels of SOCS1 expression were observed to be negatively correlated with those of tRF-23 (Figures 3C and 3D).

As an SOCS family member, SOCS1 serves as a key regulator of processes including inflammation, osteoclastogenesis, tumorigenesis, and neural stem cell proliferation/differentiation (Lafferty et al., 2014; Cui et al., 2017; Li et al., 2022; Nakatani et al., 2022). In recent years, the association between SOCS1 and osteogenic differentiation has attracted the attention of researchers. For example, the miRNA let-7e-5p can reportedly regulate MC3T3-E1 cell osteogenic differentiation via the inhibition of SOCS1, upregulating IGF-1 via the JAK2/STAT5 pathway (Wang et al., 2021a). It has been shown that miR-29b reverses osteoblastic differentiation in aortic valve interstitial cells while regulating pyroptosis via the STAT3/SOCS1 signaling pathway (Fang et al., 2023). In addition, SOCS1, the feedback regulator of STAT1/3, impairs osteogenic differentiation in rat BMSCs (Zhang et al., 2022). These findings confirm the close associations between SOCS1 and osteogenic differentiation from many aspects. The functions of SOCS1 during osteogenic differentiation of hBMSCs, however, are not fully understood. Here, SOCS1 was also confirmed to act as a key regulator of osteogenesis in hBMSCs (Figures 3E–3G), and tRF-23 was confirmed to control this osteogenic process through its ability to target SOCS1, providing a new direction for functional research focused on this regulatory protein.

While tRFs can exert regulatory functions in line with those that characterize miRNAs, these two classes of noncoding RNAs are not identical, with the core component argonaute (AGO) clearly reflecting the difference between the two. In humans, the AGO2 protein interacts with miRNAs to form the RNA-induced silencing complex (RISC) that subsequently interacts with the 3′-UTR of target mRNAs to promote their degradation or suppress translation (Elbarbary and Maquat, 2019). tRFs, in contrast, can potentially achieve this same function by interacting with one or more AGO family members (Choi et al., 2020). Consistently, proteins bound to tRF-23 were

(C) JAK2/STAT3 signaling inhibitor treatment suppressed the osteogenic differentiation of hBMSCs on day 14 relative to those in which tRF-23 was overexpressed without inhibitor treatment. Scale bars: 50 μm. Matrix mineralization and ALP activity were quantified based on absorbance. Data are presented as the mean ± SD ($n = 3$ independent experiments with two technical replicates per independent sample).
(D and E) RUNX2, OCN, and ALP levels on day 14 of hBMSC osteogenesis were detected via qPCR and western blotting. Data are presented as the mean ± SD ($n = 3$ independent experiments with two technical replicates per independent sample).
Data were analyzed using paired two-tailed Student's t tests. $^{**}p < 0.01$ was considered significant. OE, overexpression; shRNA, short hairpin RNA; T-JAK2, total JAK2; T-STAT3, total STAT3.

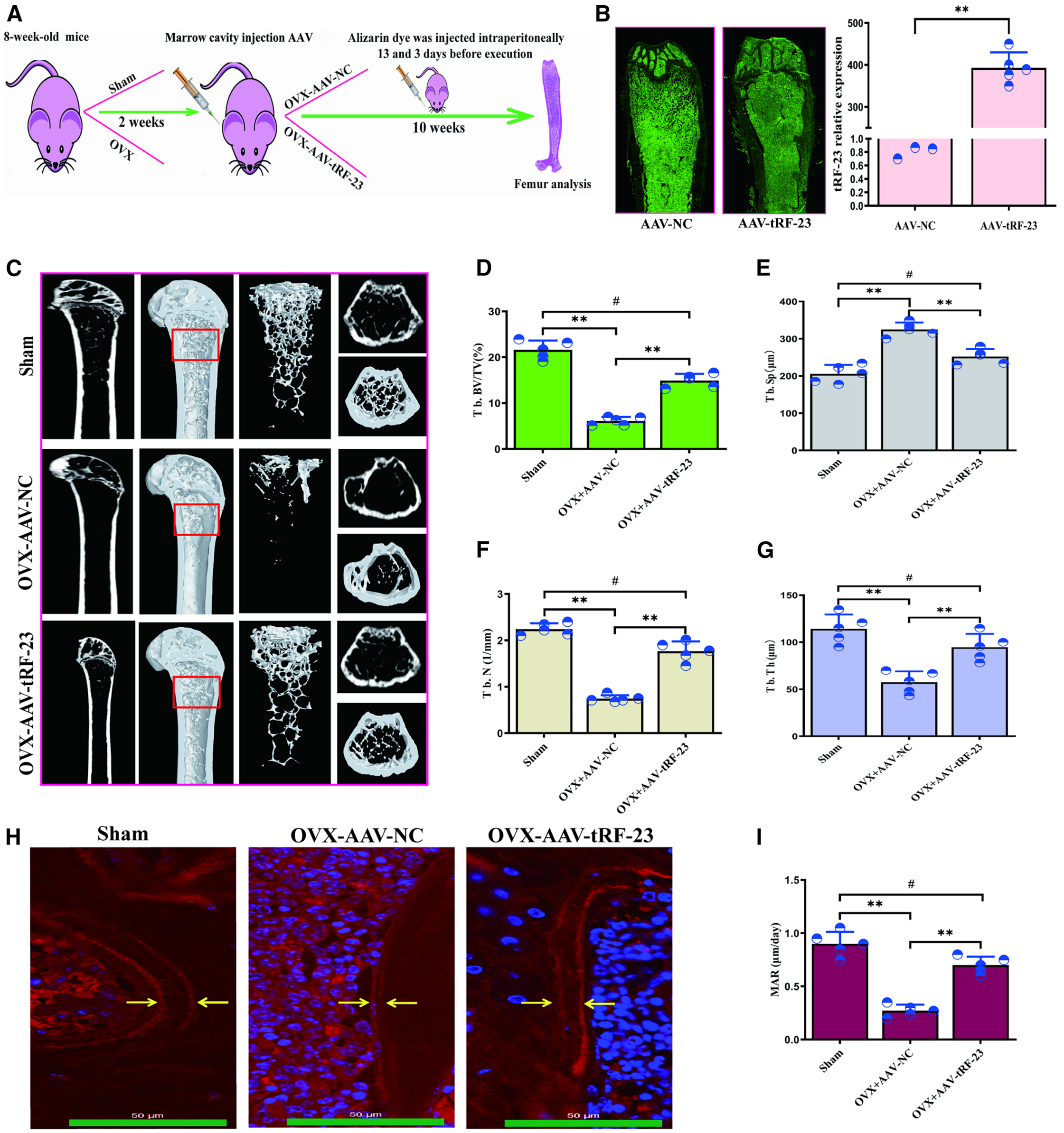


**Figure 6. Overexpressing tRF-23 in OVX mice protects against bone loss**

(A) Schematic overview of the animal experiments.

(B) The mouse femurs showed marked green fluorescence following infection with AAV, and tRF-23 expression in the femurs was analyzed. Data are presented as the mean ± SD ($n = 5$ independent experiments). Statistical analysis was performed using paired two-tailed Student's t tests. (**$p < 0.01$ was significant).

(C) Micro-CT and corresponding 3D reconstruction were used to assess femoral trabecular bone mass.

(D–G) Micro-CT analyses were used for the quantification of parameters including Tb. BV/TV, Tb.Sp, Tb.N, and Tb.Th. Data are presented as the mean ± SD ($n = 5$ independent experiments). Statistical analysis was performed using one-way ANOVA. #$p < 0.05$ and **$p < 0.01$ were considered significant.

(H and I) Representative images of Alizarin red dual staining and corresponding quantification. Scale bars: 50 μm. Data are presented as the mean ± SD ($n = 5$ independent experiments). Statistical analysis was performed using one-way ANOVA. #$p < 0.05$ and **$p < 0.01$ were considered significant. NC, negative control.

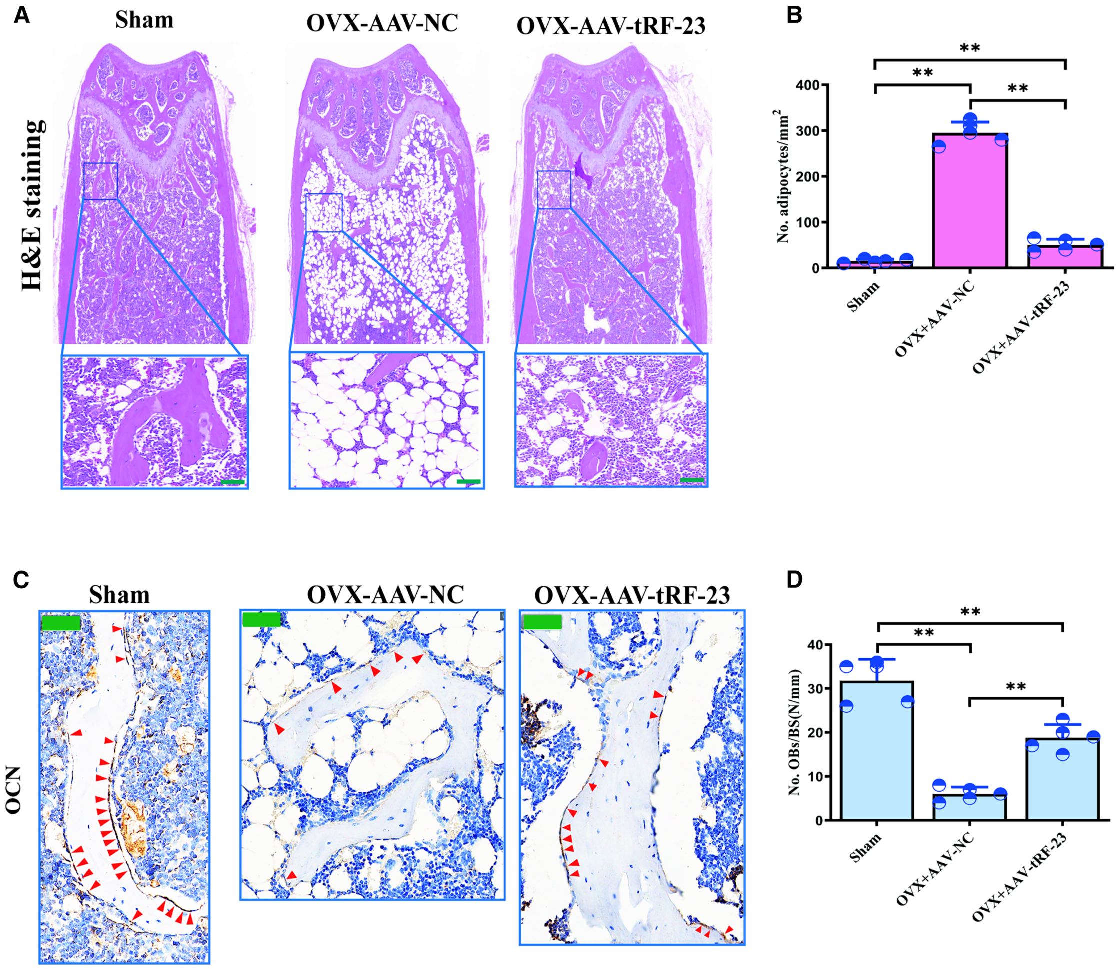


**Figure 7. Overexpressing tRF-23 in OVX mice protects against MAT accumulation and osteoblast loss**

(A and B) Representative H&E staining images and corresponding qualitative analyses of adipocyte counts per $mm^2$. Scale bars: 50 μm. Data are presented as the mean ± SD ($n$ = 5 independent experiments).

(C and D) Representative images of OCN staining-positive cells and counts of osteoblasts per mm of bone surface. Red arrowheads denote cells positive for OCN staining. Scale bars: 50 μm. Data are presented as the mean ± SD ($n$ = 5 independent experiments).

Statistical analysis was performed using one-way ANOVA. $^{**}p < 0.01$ were considered significant. NC, negative control; OB, osteoblasts; BS, bone surface.

identified by ChIRP-MS (Chu and Chang, 2018), ultimately revealing its ability to bind AGO3 (Figures 4A and 4B). RIP experiments further confirmed this interaction between AGO3 and tRF-23 (Figures 4C and 4D). The results showed that AGO3 is a major component of RISCs associated with tRF-23. When AGO3 was knocked down in cells overexpressing tRF-23 or in which SOCS1 had been knocked down, expected changes in SOCS1 and AGO3 protein levels were observed (Figures 4E and 4F). AGO3 was thus found to serve as a mediator of the targeting of SOCS1 by tRF-23 during hBMSCs osteogenic differentiation.

The JAK2/STAT3 signaling axis is closely related to cellular differentiation and was thus explored as a possible downstream target of the effects of tRF-23 on SOCS1 (Steyn et al., 2019). In addition, studies have confirmed that SOCS1 is an upstream inhibitor of the JAK2/STAT3 signaling pathway. Specifically, the SH2 domain of SOCS1 can bind to tyrosine residues in the JAK2 catalytic domain with high affinity. This inhibits the kinase activity of JAK2, thereby blocking phosphorylation of STAT3, and ultimately inducing precise regulation of the JAK2/STAT3 pathway (Wang et al., 2021b). These studies suggest further research directions for us. This SOCS1/JAK2/STAT3 pathway likely serves as an important mediator of osteogenic differentiation in hBMSCs. Overexpressing tRF-23 increased p-JAK2 and p-STAT3 levels during osteogenic

differentiation, while the opposite was observed when it was knocked down (Figures 5A and 5B), consistent with an important role for SOCS1/JAK2/STAT3 signaling in osteogenesis. JAK2/STAT3 phosphorylation was suppressed in tRF-23-overexpressing cells by treatment with AG490 (Figures 5A and 5B), resulting in reduced osteogenic differentiation of hBMSCs overexpressing tRF-23 relative to the control tRF-23-overexpressing hBMSCs (Figures 5C–5E). SOCS1/JAK2/STAT3 signaling axis is thus controlled by tRF-23, which functions as an upstream regulator. These results thus support a model wherein tRF-23 controls hBMSCs osteogenesis through its ability to target SOCS1 and alter JAK2/STAT3 signaling activity.

Given these promising *in vitro* findings, an OVX-induced murine model of osteoporosis was established, and AAV9 vectors were introduced to overexpress tRF-23 in the bone marrow in a subset of these animals. The local or systemic AAV9-mediated delivery of forkhead box O3a (FOXO3a), major vault protein, and immunoglobulin superfamily member 23, for instance, have been demonstrated to protect against administration pathologic bone loss in mice (Yuan et al., 2019, 2021; Li et al., 2020). This same approach was used to overexpress tRF-23 in this study by injecting AAV9-tRF-23 into the bone marrow, leading to a significant increase in bone trabeculae numbers in the bone marrow cavity relative to the OVX-AAV9-NC group (Figure 6C), and a corresponding reduction in vacuolar adipose cell accumulation in these treated mice (Figures 7A and 7B). As important osteoblast and adipose cell precursors, hBMSCs strictly regulate cellular fait and preserve the balance between osteogenic and adipogenic differentiation to safeguard skeletal health (Yu et al., 2018; Liu et al., 2021). tRF-23 was herein significantly found to reverse this bone-fat imbalance, as evidenced by improvements in multiple trabecular bone parameters (Figures 6D–6G). MAR and immunohistochemical analyses provided further confirmation of the ability of tRF-23 to remediate a loss of bone mass (Figures 6H, 6I, 7C, and 7D).

These *in vivo* results thus provide the first evidence that tRF-23 may be a viable target associated with the alleviation of bone loss and protection against the accumulation of MAT in a mouse OVX-induced model of osteoporosis. Even though tRF-23 treatment did not completely restore trabeculae bone density to the levels seen in the Sham group, it has important clinical reference value for the prevention and treatment of osteoporosis. Furthermore, following treatment with OVX-AAV-tRF-23, the expression levels of SOCS1, AGO3, p-JAK2, and p-STAT3 were analyzed *in vivo* (Figure S3), with results consistent with those of the *in vitro* cell experiments. The *in vivo* experiments also confirmed that tRF-23 promotes osteogenic differentiation by targeting SOCS1 via the JAK2/STAT3 signaling pathway. Here, it should be noted that although ARS and ALP staining were used in this study to evaluate osteogenic differentiation, the gold standard for such evaluation remains *in vivo* transplantation, in which cells are combined with an appropriate scaffold and implanted into immunocompromised mice. Therefore, the absence of *in vivo* transplantation data represents a minor limitation of this study, but the compelling functional outcomes from the OVX mouse model substantiate the therapeutic potential of tRF-23, effectively counterbalancing this limitation.

In summary, the results of this study demonstrate the mechanisms through which the novel non-coding small RNA tRF-23 can regulate hBMSCs osteogenesis. These findings offer a foundation for further studies of the pathogenesis of senile osteoporosis and the selection of new therapeutic targets for the management of postmenopausal osteoporosis.

## METHODS

### Cell culture and osteogenic differentiation

Flow cytometry was used to phenotype hBMSCs (HUXMA-01001, Cyagen Biosciences, China; the cell lot numbers of three donors were 150724I31, 161125R41, and 160202I31), which were validated as being ≥95% $CD73^-$, $CD90^-$, and CD105-positive, in addition to being $CD34^-$, $CD19^-$, $CD11b^-$, $CD45^-$, and HLA-DR-negative (≤5%). Cells were routinely cultured at a density of $5 \times 10^4$ cells/$cm^2$ using OriCell H-BMSCs growth media (HUXMA-90011, Cyagen Biosciences) with 10% fetal bovine serum, glutamine, and penicillin/streptomycin in a 5% $CO_2$ humidified 37°C incubator. Cells were passaged with 0.25% trypsin-ethylene diamine tetraacetic acid (Gibco; Thermo Fisher Scientific, Inc.) every 3–4 days, and cells from passages 3–6 were employed for all experiments.

Osteogenic differentiation was induced by growing cells until they were 70% confluent, followed by treatment for 14 days with 50 mM ascorbic acid, 100 nM dexamethasone, and 10 mM β-glycerophosphate (Sigma-Aldrich, MO, USA), replacing the media every third day.

### Small RNA-seq

After using TRIzol (Invitrogen) to extract cellular RNA, the quality of these RNA samples was assessed with an Agilent 2200 instrument, and a NEBNext Small RNA Library Prep Kit for Illumina was used to prepare cDNA libraries for samples with an RNA integrity number score >7.0. After ligating this RNA to 3′ and 5′ adapters, first-strand cDNA was synthesized, and index PCR was employed for index sequence and sequence adapter application. Following further library purification and quality control assessment with a Bioanalyzer 2200 (Agilent, CA, USA), 150-bp

paired-end sequencing was conducted with a HiSeq X-ten instrument (Illumina, CA, USA).

### Small RNA-seq data processing and target prediction analyses

After using Trim Galore to remove any low-quality reads and adapter sequences, the remaining sequences 12–50 nt long were aligned with miRBase (http://www.mirbase.org/). Known miRNAs were identified with BWA (Burrows-Wheeler Aligner) followed by the alignment of unmapped reads to rRNAs (https://rnacentral.org/). An in-house tRNA sequence database (using sequences from http://gtrnadb.ucsc.edu/ and https://cm.jefferson.edu/MINTbase/) was then employed for analyses of all other reads that were unmapped. Intronic sequences were initially removed, followed by the addition of CCA (Cytidine-Cytidine-Adenosine) to the end of all tRNA sequences. Ultimately, 50 genomic nt were added behind these CCA residues, and the resulting sequences were selected as possible tRFs, followed by classification with tRFdb (http://genome.bioch.virginia.edu/trfdb/) and MINTBase (https://cm.jefferson.edu/MINTbase/).

Differentially expressed tRFs during osteogenesis were identified with the EB-Seq algorithm based on the following criteria: fold change >2 or <0.5 and $p < 0.05$, FDR <0.05. The miRanda and RNAhybrid tools were used to identify predicted targets of these tRFs of interest.

### Lentiviral transfection

To overexpress and knock down tRF-23 and to knock down AGO3 and SOCS1, lentiviruses from Shanghai Genechem Co., Ltd. were utilized shRNAs targeting tRF-23, AGO3, and SOCS1 were designed (target sequences: 5′-GGCGGTGAGAGCGCCGAATCCTA-3′, 5′-CGGGAACTTCTTATTCAATTT-3′, and 5′-GGTAGCACACAACCAGGTGGC-3′). In addition, a NC shRNA was used (sequence: 5′-TTCTCCGAACGTGTCACGT-3′). The synthesized DNA oligonucleotides were respectively cloned into the pGV493 (for shRNA) and pGV280 (for overexpression)-GFP vector lentiviruses. For lentiviral transfection, hBMSCs were plated in 6-well plates ($5 \times 10^4/cm^2$) until 20%–30% confluent, followed by infection using 10 μL of appropriate lentiviral particles ($1 \times 10^8$ infectious units/mL) in complete medium containing 5 μg/mL polybrene. Following a 10-h incubation, the transfection medium was exchanged for fresh media. After a further 72-h incubation, cells were treated with 0.5 μg/mL puromycin for 48 h, followed by a 6-day screening period in which medium was refreshed every day or two.

### ARS and ALP staining

ARS staining on day 14 of osteogenic differentiation was performed with ARS solution (Cyagen Biosciences Inc., S0142) as directed. Alizarin red destaining of the cell matrix was performed with 10% cetylpyridinium chloride followed by analyses of absorbance at 560 nm in a spectrophotometer. ALP staining on day 14 of osteogenic differentiation was conducted with a kit (Beyotime Biotechnology, C3206) as directed. An ALP Assay Kit (Beyotime Biotechnology, P0321) was also used as directed for quantification.

### RT-qPCR

After extracting RNA from cells using TRIzol (Invitrogen) as aforementioned, cDNA was prepared with a Reverse Transcription Kit (Thermo, CA, USA). An SYBR Premix Ex Taq kit (Toyobo, Osaka, Japan) and an ABI Prism 7500 (Applied Biosystems) were used for qPCR analyses. The primer sequences are shown in Table S1. The $2^{-\Delta\Delta Ct}$ method was employed for the quantification of relative expression.

### Western blotting

Radioimmunoprecipitation assay buffer was used to lyse cells, and the resultant proteins (15 μg/sample) were boiled for 5 min in 5 × SDS sample buffer, followed by separation on 10% or 15% sodium dodecyl sulfate-polyacrylamide gel electrophoresis (SDS-PAGE) and transfer to polyvinylidene fluoride membranes (Millipore). After blocking for 2 h using 5% non-fat milk, blots were probed overnight with 1:1000 dilutions of rabbit antibodies from Affinity specific for RUNX2 (cat no. AF5186), OCN (cat no. DF12303), SOCS1 (cat no. AF5378), AGO3 (cat no. DF9508), p-JAK2 (cat no. AF3024), JAK2 (cat no. AF6022), p-STAT3 (cat no. AF3293), STAT3 (cat no. AF6294), and glyceraldehyde 3-phosphate dehydrogenase (cat no. AF7021). Blots were then incubated with HRP-linked anti-rabbit IgG (1:5000; Cell Signaling Technology) for 1 h, followed by enhanced chemiluminescence-based visualization (BeyoECL Plus; Beyotime Institute of Biotechnology).

### Luciferase reporter assays

WT or mutated (MUT) SOCS1 3′-UTR sequences harboring the predicted site of tRF-23 binding were inserted into the pGL3 luciferase vector, and construct identity was validated through DNA sequencing. Then, 293T cells were co-transfected with these vectors with or without tRF-23 mimics. Following a further 48-h incubation, cell luciferase activity was measured, using Renilla luciferase activity for the purposes of normalization.

### ChIRP-MS

ChIRP-MS assays enable analyses of binding interactions between RNAs and endogenous proteins. After using formaldehyde to immobilize protein-nucleic acid complexes, biotin-labeled probes and magnetic streptavidin beads were used to isolate RNA-protein complexes, with proteins then being separated and analyzed via mass spectrometry

to screen for tRF-23 binding partners. Antisense oligo probes for tRF-23 were designed, synthesized, and subjected to 3′-modification with biotin-TEG by KangChen Bio-tech (Shanghai, China). ChIRP probes were then able to specifically hybridize with cross-linked tRF-23 complexes, and streptavidin-coated magnetic beads were used to purify the tRF-23-bound chromatin complex based on the biotin-TEG modification. Purified proteins from this complex were then subjected to protease digestion to produce peptides that were analyzed via mass spectrometry. Protein identification was performed using the first and second mass spectrometric peak maps.

### RNA immunoprecipitation

A Magna RIP RNA-Binding Protein Immunoprecipitation Kit (Millipore) and anti-AGO3 (Affinity, cat no. DF9508) or IgG (Affinity, cat no. S0001) were used for RIP experiments. Briefly, after lysing hBMSCs with RIP buffer, they were combined overnight with beads coated using anti-AGO3 or IgG at 4°C. After Proteinase K (Abcam, cat no. ab281339) treatment for 1 h at 60°C, immunoprecipitated RNAs were isolated and analyzed via qRT-PCR.

### OVX modeling and AAV9 delivery

In total, 15 female specific pathogen-free C57BL/6J mice (3 months old) were randomly assigned to the sham, OVX-AAV9-NC, and OVX-AAV9-Overexpression tRF-23 groups. Animals in the two OVX groups were anesthetized and OVX to induce estrogen deficiency, while sham surgery was performed for the sham control mice to evaluate the success of OVX modeling. All mice were then allowed to recover for 2 weeks, after which mice were anesthetized and the hair near the knee joint was shaved, followed by the use of surgical scissors to separate the muscle tissue, with tweezers being used to push tendons to the left side. A 29-G insulin syringe was then inserted into the bone marrow cavity and used to inject $1 \times 10^{13}$vg/mL in 10 μL of appropriate AAVs (AAV was labeled with green fluorescence) (OBiO Technology Corp., Ltd. Shanghai, China). Overlying muscle and skin were then sutured. Mice were euthanized 12 weeks after undergoing this procedure, and the femurs of these animals were isolated and fixed for 24 h using 4% paraformaldehyde (PFA). All animal experiments were conducted according to protocols approved by the Animal Care and Use Committee of Jiujiang University.

### Micro-CT and bone histomorphometric analyses

Following model establishment, femurs from each group were collected, soft tissues were removed, and fixation with 10% neutral-buffered formalin was performed, followed by washing and soaking in PBS. Prepared bones were then transferred into a tube in the Skyscan 1176 Micro-CT instrument (Skyscan, Aartselaar, Belgium) for scanning with the following settings: 70 kV, 200 μA, 10 μm Al filter, 300-ms exposure, pixel size 8.89 μm, 2 frame averaging, and 0.4-degree rotation step through 180°. Scanning CT images were obtained at up to 9 μm resolution, and 3D reconstruction was performed. The distal femur was selected as the ROI, and trabecular bone parameters including BV/TV, Tb.N, Tb.Th, and Tb.Sp in this ROI were assessed with a constant threshold.

### Histochemical and immunohistochemical staining

After using 4% PFA to fix femurs for 24 h at 4°C, they were decalcified at the same temperature for 3 weeks in 10% EDTA (pH 7.4), followed by paraffin embedding and cutting into 4-μm sections. A standard approach was used for H&E staining of these sections, while immunohistochemical staining was performed by initially performing antigen retrieval and then blocking for 1 h at 25°C with 5% bovine serum albumin (BSA). Sections were then stained with a primary antibody against OCN (Servicebio, cat no. GB11233-100) overnight at 4°C using isotype-matched controls (rabbit IgG [GB111738-100] from Servicebio), followed by the use of an HRP-streptavidin detection system (Dako) to detect immunoreactivity. Hematoxylin was employed for nuclear counterstaining. The results (added as Figure S4) confirmed that there was no nonspecific staining in the isotype control groups, verifying the specificity of the primary antibody signals.

### Alizarin-3-methyliminodiacetic acid dual staining

Mice were intraperitoneally injected twice with 30 mg/kg alizarin-3-methyliminodiacetic acid (Sigma-Aldrich) on days 13 and 3 prior to euthanasia. After euthanasia, femurs were isolated, fixed overnight with 4% PFA, and longitudinal non-decalcified 5 μm sections were used for alizarin-3-methyliminodiacetic acid dual staining to assess the MAR in Image-Pro Plus 6.0. Four visual fields selected at random from the distal metaphysis of the femur were used to analyze trabecular bone formation, with imaging having been performed under a fluorescence microscope (Leica).

### Statistical analyses

SPSS v.16.0 (SPSS, IL, USA) was utilized for statistical testing. Data are means ± standard deviation (SD) of experiments with a minimum of three biological replicates. and were compared with one-way analysis of variance (one-way ANOVA) or Student's t tests as appropriate, using a two-tailed $p < 0.05$ as the cut-off to define significance.

## RESOURCE AVAILABILITY

### Lead contact

Further information and requests for resources and reagents should be directed to and will be fulfilled by the lead contact, Tao Wang (comwangtaocom@163.com).

### Materials availability

All the materials generated and used in this study will be available upon reasonable request.

### Data and code availability

RNA-seq data are deposited at NCBI Gene Expression Omnibus under accession number GSE306555. The proteomics data have been deposited to the Proteome Xchange Consortium with the dataset identifier PXD067728. Other relevant data are available from the corresponding author upon reasonable request.

## AUTHOR CONTRIBUTIONS

T.W., X.L., and J.X. conceived this study. H.L., W.L., L.X., and C.Z. performed cellular and molecular biology experiments, collected the data, and performed the data analysis. H.L., W.L., L.X., C.Z., and T.W. performed the animal experiments. T.W., X.L., and J.X. wrote the manuscript. All authors discussed the results and commented on the manuscript.

## ACKNOWLEDGMENTS

This work was supported by the Key Project of Jiangxi Provincial Natural Science Foundation (no. 20224ACB206011), the National Natural Science Foundation of China (no. 82360177), Key Research and Development Project of Jiujiang City, Jiangxi Province (nos. S2024ZDYFN0014 and S2024ZDYFN0009), "Xuncheng Talents" project in Jiujiang City, Jiangxi Province (no. JJXC2023071), the Natural Science Foundation of Jiangxi Province (no. 20242BAB25457), and the Basic Research Program of the Science and Technology Bureau of Jiujiang City (no. S2024KXJJ0001).

## DECLARATION OF INTERESTS

The authors declare no competing interests.

## SUPPLEMENTAL INFORMATION

Supplemental information can be found online at https://doi.org/10.1016/j.stemcr.2025.102673.

Received: March 24, 2025
Revised: September 8, 2025
Accepted: September 8, 2025
Published: October 2, 2025

## REFERENCES

Black, D.M., and Rosen, C.J. (2016). Clinical Practice. Postmenopausal Osteoporosis. N. Engl. J. Med. *374*, 254–262. https://doi.org/10.1056/NEJMcp1513724.

Choi, E.J., Ren, J., Zhang, K., Wu, W., Lee, Y.S., Lee, I., and Bao, X. (2020). The Importance of AGO 1 and 4 in Post-Transcriptional Gene Regulatory Function of tRF5-GluCTC, an Respiratory Syncytial Virus-Induced tRNA-Derived RNA Fragment. Int. J. Mol. Sci. *21*, 8766. https://doi.org/10.3390/ijms21228766.

Chu, C., and Chang, H.Y. (2018). ChIRP-MS: RNA-Directed Proteomic Discovery. Methods Mol. Biol. *1861*, 37–45. https://doi.org/10.1007/978-1-4939-8766-5_3.

Cui, M., Ma, X.L., Sun, J., He, J.Q., Shen, L., and Li, F.G. (2017). Overexpression of suppressors of cytokine signaling 1 regulate the proliferation and differentiation of rat-derived neural stem cells. Acta Histochem. *119*, 680–688. https://doi.org/10.1016/j.acthis.2017.08.003.

Elbarbary, R.A., and Maquat, L.E. (2019). Evaluating the susceptibility of AGO2-loaded microRNAs to degradation by nucleases in vitro. Methods *152*, 18–22. https://doi.org/10.1016/j.ymeth.2018.05.010.

Fang, M., Li, B., Li, X., Wang, Y., and Zhuang, Y. (2023). MicroRNA-29b regulates pyroptosis involving calcific aortic valve disease through the STAT3/SOCS1 pathway. Int. J. Cardiol. *371*, 319–328. https://doi.org/10.1016/j.ijcard.2022.08.029.

Fang, S., He, T., Jiang, J., Li, Y., and Chen, P. (2020). Osteogenic Effect of tsRNA-10277-Loaded Exosome Derived from Bone Mesenchymal Stem Cells on Steroid-Induced Osteonecrosis of the Femoral Head. Drug Des. Devel. Ther. *14*, 4579–4591. https://doi.org/10.2147/DDDT.S258024.

Gao, S., and Zhao, Y. (2023). Quality of life in postmenopausal women with osteoporosis: a systematic review and meta-analysis. Qual. Life Res. *32*, 1551–1565. https://doi.org/10.1007/s11136-022-03281-1.

Jia, Y., Tan, W., and Zhou, Y. (2020). Transfer RNA-derived small RNAs: potential applications as novel biomarkers for disease diagnosis and prognosis. Ann. Transl. Med. *8*, 1092. https://doi.org/10.21037/atm-20-2797.

Johnston, C.B., and Dagar, M. (2020). Osteoporosis in Older Adults. Med. Clin. North Am. *104*, 873–884. https://doi.org/10.1016/j.mcna.2020.06.004.

Keam, S.P., and Hutvagner, G. (2015). tRNA-Derived Fragments (tRFs): Emerging New Roles for an Ancient RNA in the Regulation of Gene Expression. Life *5*, 1638–1651. https://doi.org/10.3390/life5041638.

Kim, H.K., Yeom, J.H., and Kay, M.A. (2020). Transfer RNA-Derived Small RNAs: Another Layer of Gene Regulation and Novel Targets for Disease Therapeutics. Mol. Ther. *28*, 2340–2357. https://doi.org/10.1016/j.ymthe.2020.09.013.

Lafferty, M.K., Fantry, L., Bryant, J., Jones, O., Hammoud, D., Weitzmann, M.N., Lewis, G.K., Garzino-Demo, A., and Reid, W. (2014). Elevated suppressor of cytokine signaling-1 (SOCS-1): a mechanism for dysregulated osteoclastogenesis in HIV transgenic rats. Pathog. Dis. *71*, 81–89. https://doi.org/10.1111/2049-632X.12117.

Li, G., Zhang, H., Wu, J., Wang, A., Yang, F., Chen, B., Gao, Y., Ma, X., and Xu, Y. (2020). Hepcidin deficiency causes bone loss through interfering with the canonical Wnt/β-catenin pathway via Forkhead box O3a. J. Orthop. Translat. *23*, 67–76. https://doi.org/10.1016/j.jot.2020.03.012.

Li, X., Gong, Y., Lin, X., Lin, Q., Luo, J., Yu, T., Xu, J., Chen, L., Xu, L., and Hu, Y. (2022). Down-regulation of microRNA-155 suppressed Candida albicans induced acute lung injury by activating SOCS1 and inhibiting inflammation response. J. Microbiol. *60*, 402–410. https://doi.org/10.1007/s12275-022-1663-5.

Liu, H., Liu, S., Ji, H., Zhao, Q., Liu, Y., Hu, P., and Luo, E. (2021). An adiponectin receptor agonist promote osteogenesis via regulating

bone-fat balance. Cell Prolif. *54*, e13035. https://doi.org/10.1111/cpr.13035.

Marcellusi, A., Rotundo, M.A., Nardone, C., Sciattella, P., Gazzillo, S., Rossini, M., Barbagallo, M., Antenori, A., Valle, D., and Mennini, F.S. (2020). Osteoporosis: Economic Burden of Disease in Italy. Clin. Drug Investig. *40*, 449–458. https://doi.org/10.1007/s40261-020-00904-8.

Martinez, G., Choudury, S.G., and Slotkin, R.K. (2017). tRNA-derived small RNAs target transposable element transcripts. Nucleic Acids Res. *45*, 5142–5152. https://doi.org/10.1093/nar/gkx103.

Nakatani, K., Serada, S., Fujimoto, M., Obata, K., Ohkawara, T., Sasabe, E., Yamamoto, T., and Naka, T. (2022). Gene therapy with SOCS1 induces potent preclinical antitumor activities in oral squamous cell carcinoma. J. Oral Pathol. Med. *51*, 126–133. https://doi.org/10.1111/jop.13268.

Rachner, T.D., Khosla, S., and Hofbauer, L.C. (2011). Osteoporosis: now and the future. Lancet *377*, 1276–1287. https://doi.org/10.1016/S0140-6736(10)62349-5.

Schorn, A.J., Gutbrod, M.J., LeBlanc, C., and Martienssen, R. (2017). LTR-Retrotransposon Control by tRNA-Derived Small RNAs. Cell *170*, 61–71.e11. https://doi.org/10.1016/j.cell.2017.06.013.

Steyn, P.J., Dzobo, K., Smith, R.I., and Myburgh, K.H. (2019). Interleukin-6 Induces Myogenic Differentiation via JAK2-STAT3 Signaling in Mouse C2C12 Myoblast Cell Line and Primary Human Myoblasts. Int. J. Mol. Sci. *20*, 5273. https://doi.org/10.3390/ijms20215273.

Su, P., Tian, Y., Yang, C., Ma, X., Wang, X., Pei, J., and Qian, A. (2018). Mesenchymal Stem Cell Migration during Bone Formation and Bone Diseases Therapy. Int. J. Mol. Sci. *19*, 2343. https://doi.org/10.3390/ijms19082343.

Venkatesh, T., Suresh, P.S., and Tsutsumi, R. (2016). tRFs: miRNAs in disguise. Gene *579*, 133–138. https://doi.org/10.1016/j.gene.2015.12.058.

Wang, T., Mei, J., Li, X., Xu, X., Ma, B., and Li, W. (2020). A novel tsRNA-16902 regulating the adipogenic differentiation of human bone marrow mesenchymal stem cells. Stem Cell Res. Ther. *11*, 365. https://doi.org/10.1186/s13287-020-01882-6.

Wang, C., Liu, S., Li, J., Cheng, Y., Wang, Z., Feng, T., Lu, G., Wang, S., Song, J., Xia, P., and Hao, L. (2021a). Biological Functions of Let-7e-5p in Promoting the Differentiation of MC3T3-E1 Cells. Front. Cell Dev. Biol. *9*, 671170. https://doi.org/10.3389/fcell.2021.671170.

Wang, Y., Kong, X.Q., Wu, F., Xu, B., Bao, D.J., Cheng, C.D., Wei, X. P., Dong, Y.F., and Niu, C.S. (2021b). SOCS1/JAK2/STAT3 axis regulates early brain injury induced by subarachnoid hemorrhage via inflammatory responses. Neural Regen. Res. *16*, 2453–2464. https://doi.org/10.4103/1673-5374.313049.

Weng, Q., Wang, Y., Xie, Y., Yu, X., Zhang, S., Ge, J., Li, Z., Ye, G., and Guo, J. (2022). Extracellular vesicles-associated tRNA-derived fragments (tRFs): biogenesis, biological functions, and their role as potential biomarkers in human diseases. J. Mol. Med (Berl). *100*, 679–695. https://doi.org/10.1007/s00109-022-02189-0.

Xia, H., Gao, M., Chen, J., Huang, G., Xiang, X., Wang, Y., Huang, Z., Li, Y., Su, S., Zhao, Z., et al. (2022). M1 macrophage-derived extracellular vesicle containing tsRNA-5006c promotes osteogenic differentiation of aortic valve interstitial cells through regulating mitophagy. PeerJ *10*, e14307. https://doi.org/10.7717/peerj.14307.

Xia, S.L., Ma, Z.Y., Wang, B., Gao, F., Guo, S.Y., and Chen, X.H. (2023). Icariin promotes the proliferation and osteogenic differentiation of bone-derived mesenchymal stem cells in patients with osteoporosis and T2DM by upregulating GLI-1. J. Orthop. Surg. Res. *18*, 500. https://doi.org/10.1186/s13018-023-03998-w.

Yu, B., Huo, L., Liu, Y., Deng, P., Szymanski, J., Li, J., Luo, X., Hong, C., Lin, J., and Wang, C.Y. (2018). PGC-1α Controls Skeletal Stem Cell Fate and Bone-Fat Balance in Osteoporosis and Skeletal Aging by Inducing TAZ. Cell. Stem. Cell. *23*, 615–623. https://doi.org/10.1016/j.stem.2018.09.001.

Yuan, L., Zhao, N., Wang, J., Liu, Y., Meng, L., Guo, S., Wiemer, E.A. C., Chen, Q., Mao, Y., Ben, J., and Ma, J. (2021). Major vault protein (MVP) negatively regulates osteoclastogenesis via calcineurin-NFATc1 pathway inhibition. Theranostics *11*, 7247–7261. https://doi.org/10.7150/thno.58468.

Yuan, Y., Yang, L., Liu, T., Zhang, H., and Lu, Q. (2019). Osteoclastogenesis inhibition by mutated IGSF23 results in human osteopetrosis. Cell Prolif. *52*, e12693. https://doi.org/10.1111/cpr.12693.

Zeng, L., Zhou, J., Zhang, Y., Wang, X., Wang, M., and Su, P. (2021). Differential Expression Profiles and Potential Intergenerational Functions of tRNA-Derived Small RNAs in Mice After Cadmium Exposure. Front. Cell Dev. Biol. *9*, 791784. https://doi.org/10.3389/fcell.2021.791784.

Zhang, S., Yu, X., Xie, Y., Ye, G., and Guo, J. (2023). tRNA derived fragments:A novel player in gene regulation and applications in cancer. Front. Oncol. *13*, 1063930. https://doi.org/10.3389/fonc.2023.1063930.

Zhang, Y., Jing, Z., Cao, X., Wei, Q., He, W., Zhang, N., Liu, Y., Yuan, Q., Zhuang, Z., Dong, Y., et al. (2022). SOCS1, the feedback regulator of STAT1/3, inhibits the osteogenic differentiation of rat bone marrow mesenchymal stem cells. Gene *821*, 146190. https://doi.org/10.1016/j.gene.2022.146190.

Zhou, M., He, X., Zhang, J., Mei, C., Zhong, B., and Ou, C. (2024). tRNA-derived small RNAs in human cancers: roles, mechanisms, and clinical application. Mol. Cancer *23*, 76. https://doi.org/10.1186/s12943-024-01992-2.

**Stem Cell Reports, Volume *20***

## Supplemental Information

# The novel tRF-23 promotes osteogenic differentiation of hBMSCs and protects against bone loss in ovariectomized mice

**Haichun Liao, Wen Li, Lin Xu, Chao Zhao, Xingnuan Li, Jianjun Xiong, and Tao Wang**

# Supplemental Information

## The novel tRF-23 promotes osteogenic differentiation of hBMSCs and protects against bone loss in ovariectomized mice

**Haichun Liao, Wen Li, Lin Xu,**

**Chao Zhao, Xingnuan Li, Jianjun Xiong, and Tao Wang**

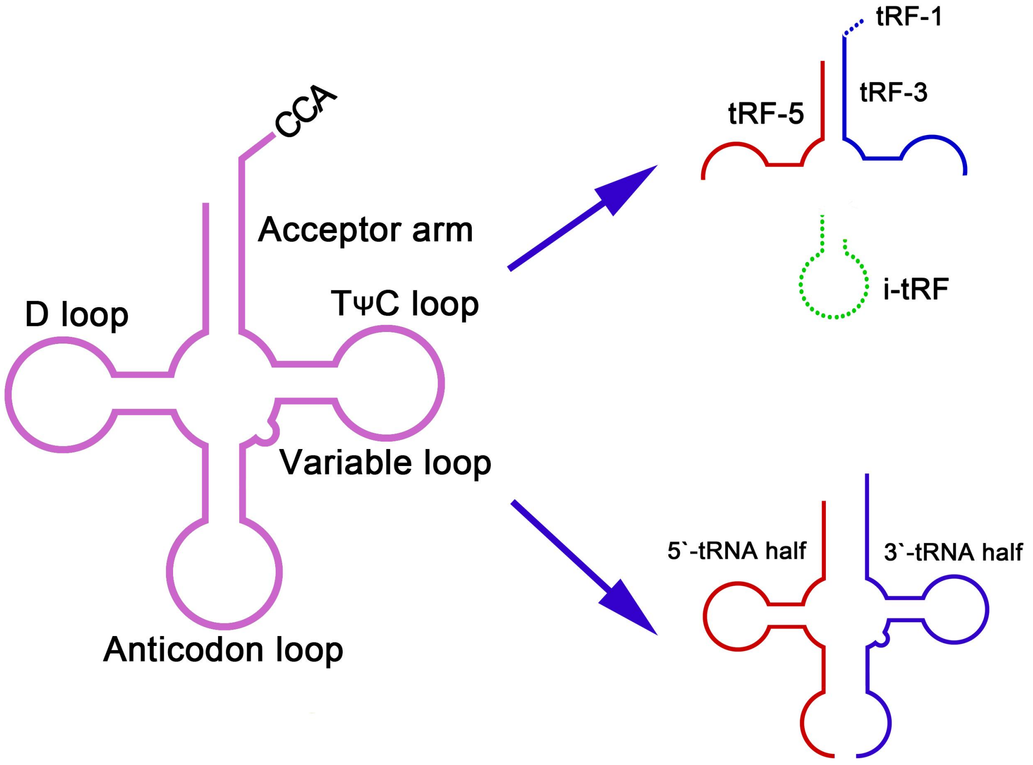


**Figure S1. Different tRFs are derived from pre-tRNAs or mature tRNAs, related to Figure 1**

tRFs are classified as tRF-5 and tRF-3, i-tRFs, tRF-1, and 5′- or 3′-tRNA halves based on their compositions and origins.

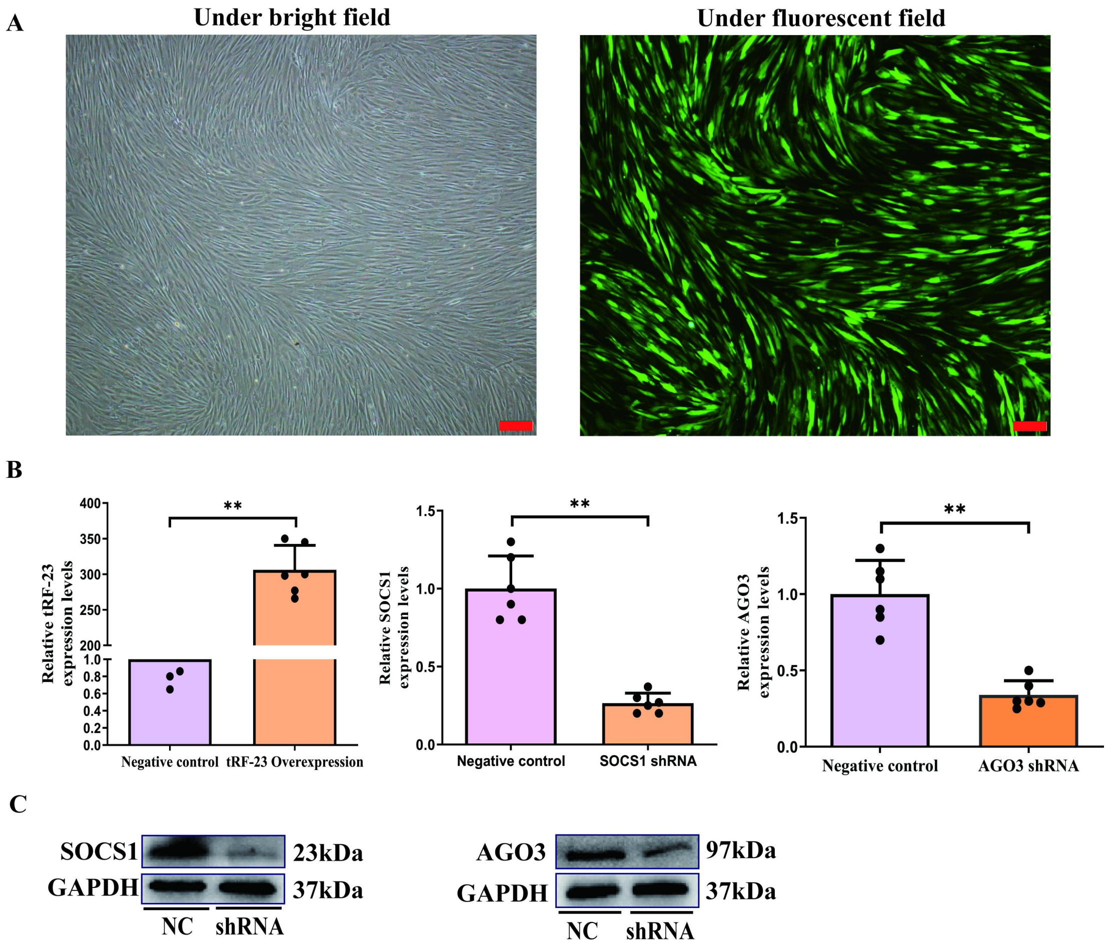


**Figure S2. Efficiency of lentiviral transfection at 6 days post-transfection**

(A) Transfected cells were evaluated using fluorescence and light microscopy, with representative images shown. Scale bar: 100 μm.

(B and C) qPCR and Western immunoblotting were used to evaluate tRF-23, SOCS1, and AGO3 expression after lentiviral transfection. Data are presented as the mean ± SD (n = 3 independent experiments with two technical replicates per independent sample).

Statistical analysis was performed using a two-tailed paired Student's t test. $^{**}P < 0.01$ was considered significant. Note: NC, negative control; shRNA, short hairpin RNA.

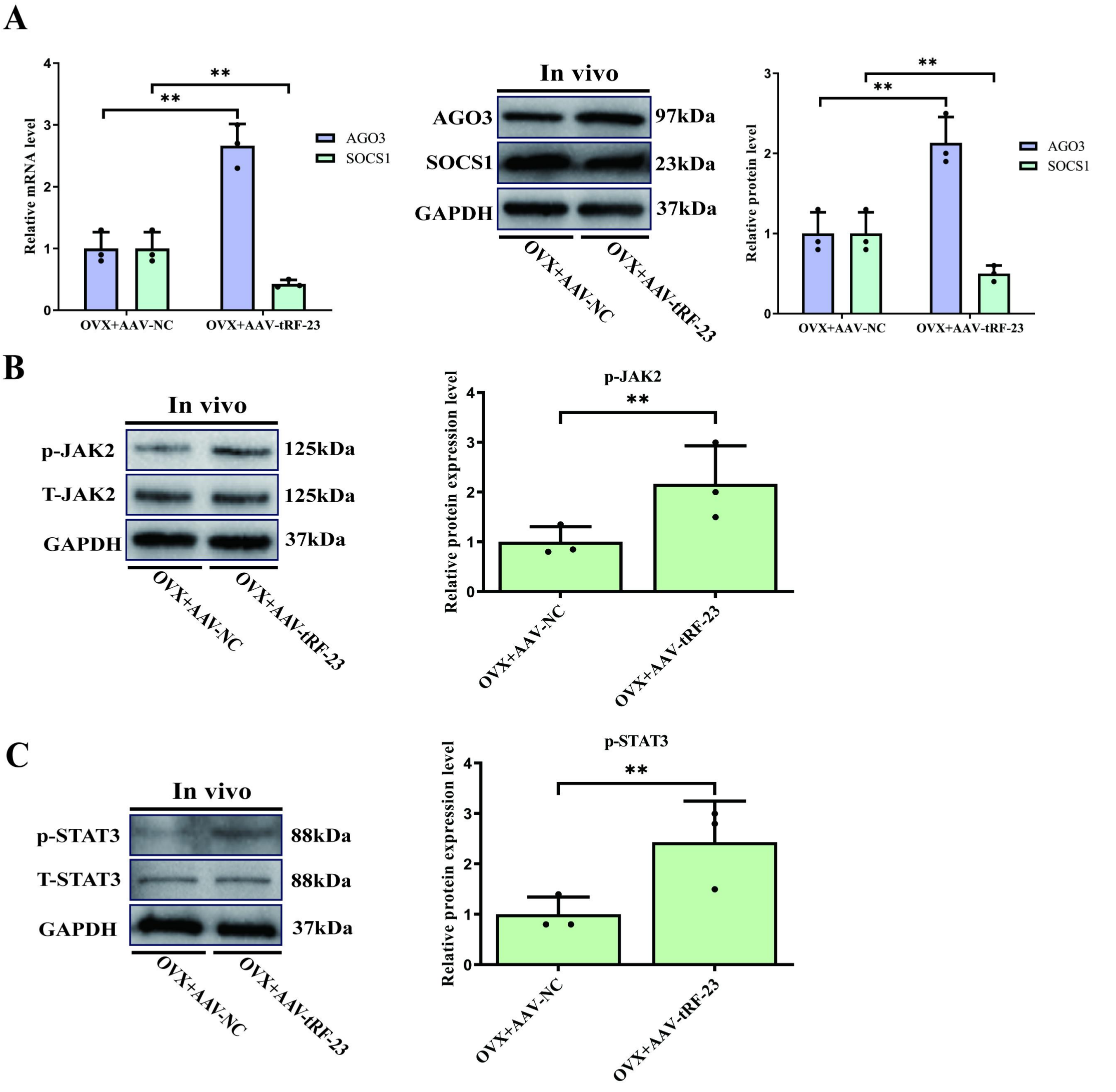


**Figure S3. The effects of intramural injection of AAV9-tRF-23 on the expression of SOCS1, AGO3, and components of the JAK2/STAT3 signaling pathway in mouse femur, related to Figures 6 and 7**

(A)Reduced expression of SOCS1 in the OVX-AAV-tRF-23 mouse model, with increased expression of AGO3 compared with the OVX-AAV-NC group. Data are presented as the mean ± SD (n = 3 independent experiments).

(B and C) AVV-tRF-23 administration to OVX model mice induced increased levels of p-JAK2/STAT3 proteins compared with the OVX-AAV-NC group. Data are presented as the mean ± SD (n = 3 independent experiments).

Statistical analysis was performed using a two-tailed paired Student's t test. ***P* < 0.01 was considered significant. Note: NC, negative control.

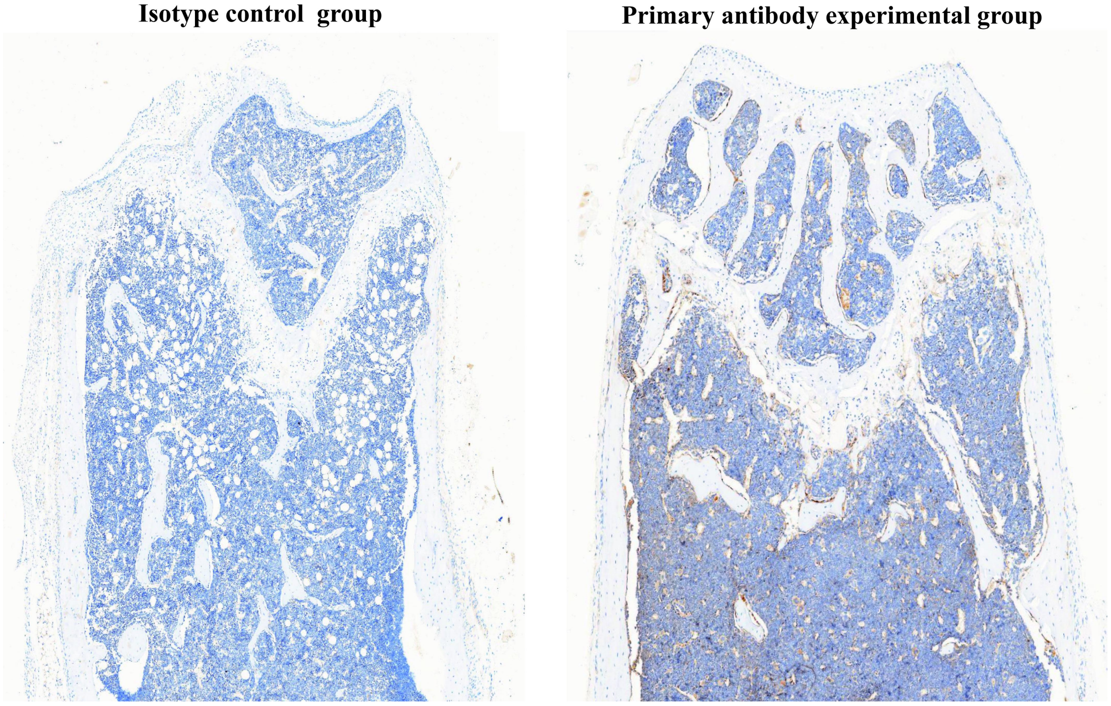


**Figure S4. Non-immune immunoglobulins of the same isotype as the primary antibodies used as negative controls, related to Figure 7**

The results confirmed that there was no nonspecific staining in the isotype control groups, verifying the specificity of the primary antibody signals.

**Table S1. Primer sets used in this study.**

| Gene symbol | Forward primers | Reverse primers |
|---|---|---|
| RUNX2 | 5′-GGACGAGGCAAGAGTTTCACC-3′ | 5′-GGTTCCCGAGGTCCATCTACT-3′ |
| OCN | 5′-TGAGAGCCCTCACACTCCTC-3′ | 5′-CGCCTGGGTCTCTTCACTAC-3′ |
| ALP | 5′-CCCCGTGGCAACTCTATCTTT-3′ | 5′-GCCTGGTAGTTGTTGTGAGCATAG-3′ |
| SOCS1 | 5′-GAGCTGCTGGAGCACTACG-3′ | 5′-AGGGGAAGGAGCTCAGGTAG-3′ |
| AGO3 | 5′-ACATGCGAGGGAAACAATTC-3′ | 5′-ACATGGGCTCTACGCTGTCT-3′ |
| β-actin | 5′-GCGAGAAGATGACCCAGATCATGT-3′ | 5′-TACCCCTCGTAGATGGGCACA-3′ |
| tRF-23 | 5′-CTCAACTGGTGTCGTGGAGTCGGCAATTCA GTTGAGGGCGGTGA-3′ | 5′-ACACTCCAGCTGGGTAGGATTCGGCGCTCTC-3′ |
| U6 | 5′-CTCGCTTCGGCAGCACA-3′ | 5′-AACGCTTCACGAATTTGCGT-3′ |
